# Modelling irregular nonlinear waves: potential flow and volume-of-fluid comparison with experiment

**DOI:** 10.1007/s40722-026-00495-0

**Published:** 2026-07-11

**Authors:** Samuel Draycott, Xiang Li, Qing Xiao, Peter Stansby

**Affiliations:** 1 School of Engineering, University of Manchester, Manchester, UK; 2 Department of Naval Architecture, Ocean and Marine Engineering, University of Strathclyde, Glasgow, UK

**Keywords:** Irregular waves, Experimental testing, Numerical modelling, Potential flow, Computational fluid dynamics, Wave breaking

## Abstract

Real ocean wave conditions are irregular and often highly nonlinear, frequently involving wave breaking. As such, numerical methods to recreate these conditions are critical for the de-risking of offshore structures and devices installed in these environments. There are a range of numerical tools available, with varying fidelity and associated computational expense: from potential flow models to two-phase computational fluid dynamics (CFD) simulations able to resolve breaking physics. Here we present comparisons between the fully nonlinear potential flow model OceanWave3D, and the finite volume OpenFOAM solver with volume-of-fluid to capture the air–water interface, for the recreation of irregular wave conditions. A series of eleven well-defined unidirectional irregular conditions, including breaking conditions, are conducted in the FloWave Ocean Energy Research Facility, and equivalent wave generation inputs are provided to both OceanWave3D and OpenFOAM. Statistical and spectral comparisons demonstrate that both models reproduce surface elevations and wave kinematics well, with only OpenFOAM capturing spilling breaking events. OpenFOAM’s higher fidelity comes at significantly increased computational cost. The run times are $\mathcal {O}$(mins) for OceanWave3D and $\mathcal {O}$(days) for OpenFOAM and hence it is concluded that the optimal approach depends on the importance of capturing breaking events accurately for the given application, and on the size of domains required.

## Introduction

Offshore structures must be designed to withstand realistic ocean wave conditions, which are invariably irregular, nonlinear, and often involve wave breaking. Accurate prediction of these conditions up to the limiting steepness is, therefore, required for effective design. When breaking is initiated, this substantially increases complexity whereby the crest overturns and induces turbulence and air entrainment. There are two main approaches for modelling nonlinear waves with treatment of wave breaking: fully nonlinear potential flow (FNPF) models with breaking identification and local dissipation, and Navier–Stokes solvers where typically a two-phase (water/air) volume-of-fluid approach is used to capture the interface. The latter is often referred to as computational fluid dynamics (CFD). Practically, there are established codes for both FNPF and CFD, with OceanWave3D (Engsig-Karup et al. 2009), REEF3D-FNPF (Bihs et al. 2020), and HOS-NWT (and HOS-Ocean) (Bonnefoy et al. 2010; Ducrozet et al. 2016) being commonly used FNPF codes and OpenFOAM (Weller et al. 1998), Ansys CFX (ANSYS 2006) and STAR CCM+ (Siemens Digital Industries Software 2021) amongst common CFD codes. The above codes mentioned are all open source apart from Ansys CFX and STAR CCMred+, and here we consider the long established OceanWave3D (hereafter OW3D) and OpenFOAM codes.

In terms of validation and comparison to experiment, FNPF codes have been validated against experiment for a wide range of cases: regular waves (e.g. Engsig-Karup et al. (2009); Bihs et al. (2020)), focused waves (e.g. Ghadirian et al. (2016); Barratt et al. (2020); McAllister et al. (2022)), irregular unidirectional waves (e.g. Li et al. (2023b); Draycott et al. (2022)), and even directionally spread irregular wave fields (e.g. Tang et al. (2023)). For CFD codes, however, there are various works focusing on regular waves (e.g. Chen et al. (2014); King et al. (2023)) and unidirectional focused wave groups (e.g. Ransley et al. (2021)), yet there is remarkably little validation for irregular waves, in large part due to the computational expense associated with simulating these conditions in CFD codes. A few notable exceptions exist (Jensen et al. 2014; Jacobsen et al. 2015; Paolo et al. 2021), whereby OpenFOAM was compared to experiments for the interaction of irregular waves with coastal structures, but the analysis is complicated by the presence of the structures themselves, and there are limited phase-resolved comparison of the generated waves.

A number of studies comparing or combining FNPF and CFD solvers have been reported, often in the context of ship seakeeping and wave–structure interaction. Examples include direct comparisons between FPNF and CFD predictions of motions and added resistance (Sulovsky et al. 2023), hybrid approaches where potential flow solutions are modified or corrected using CFD information (Nam et al. 2022), or zonal coupling between FNPF and CFD codes for wave-structure interaction, e.g. Li et al. (2018). Previous studies typically focus on regular or focused waves and on structural response, rather than on phase-resolved irregular sea states in the absence of structures.

The aim of this paper is, therefore, to assess the performance of well-known and highly used FNPF and CFD codes for the phase-resolved generation of short-run unidirectional irregular wave fields. Analysis is conducted in the absence of any structure to understand the underlying differences in the generated wave fields. Both OpenFOAM and OceanWave3D numerical wave tanks along with a physical wave basin are given the same input wave specification without any corrections applied to understand the underlying differences and implications of using one of these methods to simulate irregular waves. Experimental measurements of surface elevations from the circular FloWave basin, with reflections removed, are compared to numerical model outputs. To enable direct and fair comparison, both the experimental wavemakers and numerical wavemakers are given effectively the same input, with a specified set of wave components with defined frequencies, amplitudes, and phases, designed to give a target linear time series at a chosen location (12.5 m from wavemakers) using linear wave theory. Comparisons are made over 11 wave cases with varying significant wave height $H_{m0}$ and peak period $T_p$, including limiting steepness cases with spilling breaking waves.

The structure of the paper is as follows. In Sect. 2, the experimental and numerical setups are defined. Results are presented in Sect. 3 including statistical and spectral comparisons of surface elevations between experiment and models, and inter-model comparisons of kinematics, dispersion, and dissipation. A further discussion on model strengths and limitations is presented in Sect. 4 before concluding remarks are offered in Sect. 5.

## Experimental and numerical setup

### Wave conditions and wave input

Eleven irregular wave cases were specified based on target JONSWAP energy density spectra, *S*(*f*), (Hasselmann et al. 1973). As detailed in Table 1, these cases have different values of significant wave height, $H_{m0}$, and peak period, $T_p$, with a constant peak enhancement factor, γ = 3.3. This results in a range of sea state steepness values, $0.5 H_{m0} k_p$, as detailed in Table 1.

In order to define the linear conditions precisely, discrete frequency components, $f_j$, are required with an associated amplitude, $a_j$, and phase, $\phi _j$, defined at some target location, $x_{\textrm{tar}}$. The desired complex linear amplitude spectrum at the target location is, therefore, defined as:

1 $$\begin{aligned} A_{j} = a_j e^{i\phi _j} \end{aligned}$$

where $a_j = \sqrt{2S(f_j)\Delta f}$ and, for irregular sea states $\phi _j$ are random phases which are uniformly distributed between 0 and 2π. The discrete frequency increments, $\Delta f = f_{j+1}-f_j$, are determined based on the sea state repeat time $T_r$, where $\Delta f = 1/T_r$. For all conditions, $T_r$ is set to 64 s with the run time $T_{\textrm{run}}$ set to 100 s providing time for all wave components to arrive at the measurement locations. The frequencies are defined between 0.5$f_p$ and 2.5$f_p$ where $f_p$ is the peak frequency. The final 64 s (36–100 s) are used for analysis in order to give a full repeat time of measurement to maximise stationarity. Reflections that accumulate over the course of the experiment prevent the wave field from ever becoming fully stationary. Over the $64~\textrm{s}$ analysis window, this corresponds to approximately $64/T_p$ peak-wave cycles, i.e. ∼53 cycles for $T_p=1.2~\textrm{s}$, ∼46 cycles for $T_p=1.4~\textrm{s}$, and ∼36 cycles for $T_p=1.8~\textrm{s}$.

**Table 1:** Target wave conditions. All conditions have JONSWAP peak enhancement factor γ = 3.3

ID	1	2	3	4	5	6	7	8	9	10	11
$H_{m0}$ (m)	0.06	0.06	0.06	0.09	0.09	0.09	0.13	0.13	0.13	0.16	0.16
$T_p$ (s)	1.2	1.4	1.8	1.2	1.4	1.8	1.2	1.4	1.8	1.4	1.8
γ	3.3	3.3	3.3	3.3	3.3	3.3	3.3	3.3	3.3	3.3	3.3
$0.5 H_{m0}k_p$	0.083	0.062	0.039	0.12	0.094	0.058	0.18	0.14	0.084	0.17	0.10

In order to generate waves in both physical and numerical wave basins, it is required to define the wave field at the boundary. When specific target phases ($\phi _j$) are defined away from the wavemaker at a target location $x_{tar}$, it is common to assume linear dispersion in order to obtain the phases at the boundary required for wave generation. The complex linear amplitude spectrum at the boundary position, $x_b$, can therefore be calculated using:

2 $$\begin{aligned} A_{b,j} = a_j e^{i[\phi _j -k_j (x_b-x_{\textrm{tar}})]} = A_j e^{-i k_j (x_b-x_{\textrm{tar}})} \end{aligned}$$

where $k_j$ is the wavenumber associated with frequency component *j* and is related through the linear dispersion relation for arbitrary depth:

3 $$\begin{aligned} \omega _j = \sqrt{g k_j \tanh {(k_j h)}} \end{aligned}$$

where $\omega _j = 2 \pi f_j$, *h* is the water depth and *g* is acceleration due to gravity.

For OW3D, an Inverse Fast Fourier Transform (IFFT) is applied to $A_{b,j}$ in Eq. 2 in order to define the desired linear surface-elevation time-series which is imposed in the relaxation zone.

For OpenFOAM, we use a hinged paddle equivalent to the paddles in the FloWave basin (see Section 2.2). We define a linear transfer function relating surface-elevation amplitude Fourier coefficients to Fourier coefficients for the amplitude of the motion of the paddle at the surface (as in Schäffer (1996); Kusumawinahyu et al. (2017)):

4 $$\begin{aligned} T_{b,j} = \frac{\left( \sinh {k_jh}\cosh {k_jh}+k_jh \right) \left( \frac{k_j(h-h_0)}{2}\right) }{\sinh {k_jh}\left[ (h-h_0)k_j\sinh {k_jh}-\cosh {k_jh}+\cosh {k_jh_0}\right] } \end{aligned}$$

where $h_0 = h-h_{\textrm{hinge}}$ is the distance from the floor to the wavemaker hinge and $h_{\textrm{hinge}}$ is the submerged length of the wavemaker. We then convert this transfer function to angle:

5 $$\begin{aligned} T_{\theta ,b,j} = \arctan {{\left( \frac{T_{b,j}}{(h-h_0)}\right) }} \end{aligned}$$

Finally the wavemaker hinge angle Fourier coefficients are computed as:

6 $$\begin{aligned} \theta _{b,j} = -i \ A_{b,j} \ T_{\theta ,b,j} \end{aligned}$$

and an IFFT is used to calculate the time series of wavemaker angular motion which is imposed in OpenFOAM.

Note that in the laboratory, we effectively provide the surface-elevation amplitude coefficients at the target position $A_j$ (Eq. 1) to the software; however, a basically identical process is conducted in the wave generation software: applying linear transfer functions to convert the complex elevation amplitude spectra (Eq. 2) to paddle torque, before taking the IFFT to create time-domain target torque signals.

Where possible, we configure each numerical wave tank to match the FloWave experimental setup (effective 2D geometry, depth, target location, and wavemaker characteristics), noting that perfect equivalence is not achievable because OW3D and OpenFOAM employ fundamentally different wave generation and dissipation mechanisms. The slightly different generation approaches used across the experiments, OW3D and OpenFOAM, can affect phase (effective generation distance and transfer function phase), high-frequency content (flap-type wavemaking can introduce free/error-wave components and higher harmonics), and subsequent nonlinear evolution (linear phase back-propagation becomes less accurate for short-period/steep cases and, in FloWave, is further complicated by the circular geometry). We, therefore, attribute the largest phase offsets to a combination of flap transfer function/free-wave effects (experiment and OpenFOAM) and nonlinear evolution between the generation region and the target location (all methods), with the relative contribution differing across systems.

### Experimental setup and approach

Experiments were conducted at the FloWave Ocean Energy Research Facility at the University of Edinburgh, UK., which has a unique circular geometry enabling the generation of complex directional wave fields (e.g. McAllister et al. (2019, 2024)) and combined wave-current conditions (e.g. Draycott et al. (2018b, 2019)). The FloWave basin is 2 m deep with a diameter of 25 m, and is encircled by 168 flap-type force-feedback wavemakers with a hinge depth $h_{\textrm{hinge}} = 1.7$ m. In this, study, however, we limit the conditions to only unidirectional wave conditions in the absence of current, as specified in Sect. 2.1.

A series of 11 resistance-type multiplexed wave gauges were installed along the tank centreline (y=0) to measure surface elevations, spanning a total length of 12 m as detailed in Table 2. Typical errors associated with these wave gauges (including calibration error) are less than 0.5 mm (McAllister et al. 2019; HR Wallingford 2018). Wave gauges 4–8 were designed to facilitate reflection analysis, with spacing based on Golomb ruler to guarantee an evenly spaced co-array (see Draycott et al. (2018a)).

**Table 2:** FloWave wave gauge placement relative to target position for both short and long runs. The target position for phase definition is at wave gauge 6 (*x* = 0), which is 12.5 m from the wavemakers

Gauge number	1	2	3	4	5	6	7	8	9	10	11
*x* (m)	-6	-4	-2	−0.36	−0.27	0	0.45	0.64	2	4	6
*y* (m)	0	0	0	0	0	0	0	0	0	0	0

#### Removing reflections

To enable the fairest comparisons between the experiments and the numerical models (Sects. 2.3 and 2.4), we seek to remove reflections arising from imperfect wave absorption. These reflections are present in the FloWave measurements (see, e.g. Draycott et al. (2016); Draycott (2017)) and can bias comparisons if left untreated. In the numerical wave tanks, reflections are reduced by the applied absorption treatments but are not strictly zero. Their magnitude is quantified separately in Appendix A.

Reflections in the experimental records are separated using the linear frequency-domain method of Zelt and Skjelbreia (1993). The method assumes linear wave theory and therefore provides only an approximate decomposition for the steepest cases, where nonlinear bound-wave content and breaking will violate the underlying assumptions. Nevertheless, it provides a more appropriate experimental reference signal than the raw measurements due to the magnitude of the reflections (see Appendix A). Unless stated otherwise, comparisons in this paper, therefore, use the separated incident component of the experimental surface elevation.

This reflection-separated experimental signal is used as the reference for the comparisons that follow, noting that the separation is approximate for the steepest cases. We do not apply the same separation to the numerical outputs in the primary comparisons because linear decomposition can misattribute nonlinear bound-wave content as reflected energy, particularly for the steepest cases, and because residual reflections in the numerical wave tanks are part of the realised wave field produced by each numerical wave-tank configuration. In addition, a consistent reflection separation of kinematics would also be challenging and is not appropriate in breaking conditions. To test the sensitivity of the results and conclusions of this decision, Appendix A provides statistical comparisons (coefficients of determination $r^2$) for all cases when reflections have been removed from experiments and models comparably, confirming consistency and that conclusions remain unchanged. It is worth acknowledging here, that whilst the FloWave incident measurements provide the most direct reference for the realised wave field, this reference data remains subject to residual uncertainty due to imperfect incident–reflected decomposition in steep/breaking conditions, finite wave-gauge accuracy (albeit small expected errors see Section 2.2), and facility-specific effects (e.g. weak re-reflections and basin geometry).

**Fig. 1:**
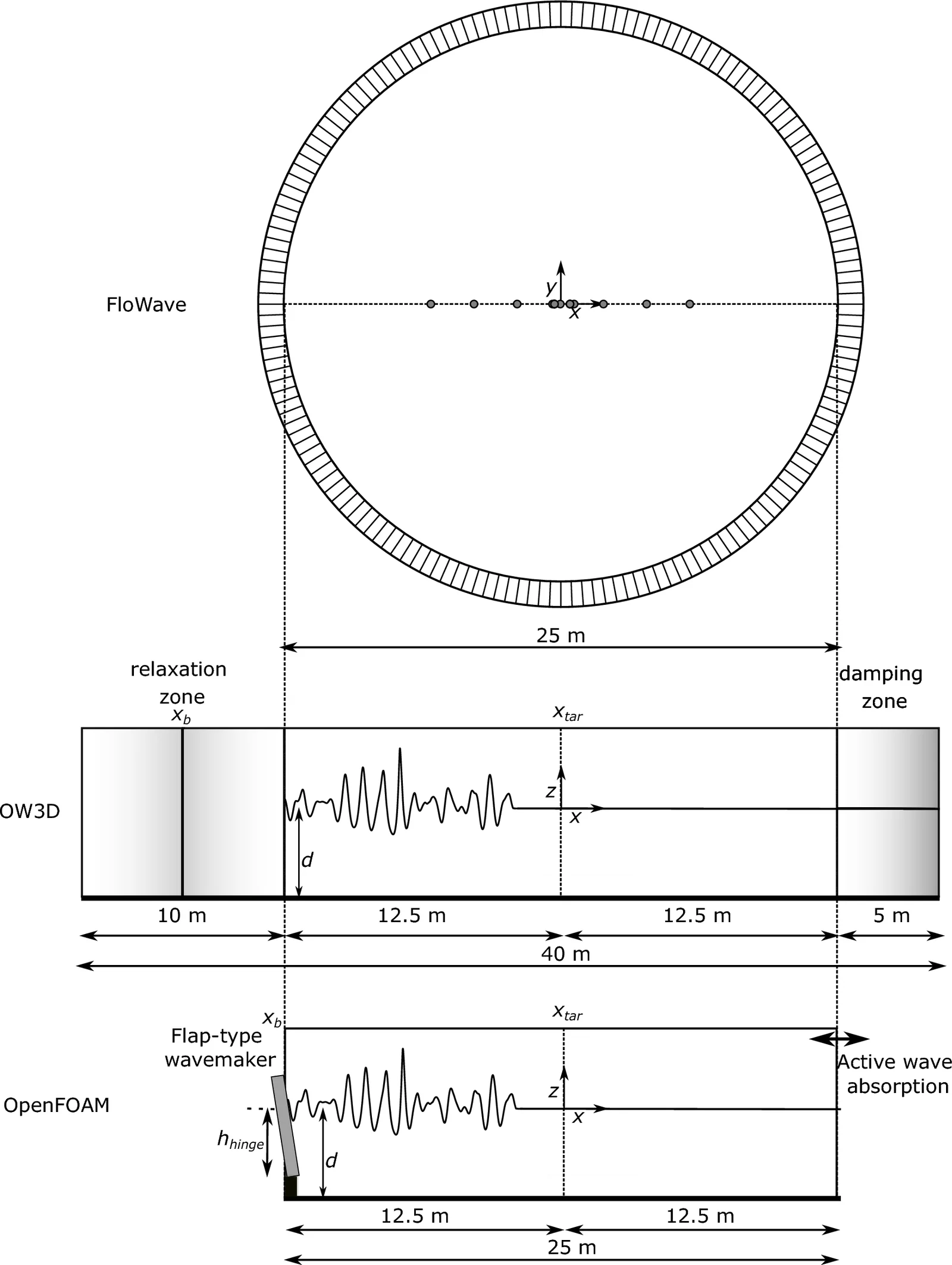
Diagram of the FloWave basin in plan view (top), the OW3D numerical flume in elevation view (middle), and the OpenFOAM numerical wave flume in elevation view (bottom). Numerical flumes are set up to be 2D representations of the 3D FloWave tank

### OceanWave3D

OceanWave3D (Engsig-Karup et al. 2009) is a FNPF model which solves the Laplace equation subject to the fully nonlinear dynamic and kinematic boundary conditions on the free water surface, and the kinematic boundary condition on the solid (in this instance, flat) bed. Computations are carried out in a two-dimensional Eulerian frame with $\sigma _z$-coordinate system, time integration is conducted using a fourth-order Runge–Kutta scheme, and spatial derivatives are calculated using a sixth-order finite differencing scheme (Ducrozet et al. 2010).

The model was set up to effectively be a 2D representation (single node in the *y* direction) of the FloWave wave basins as depicted in Fig. 1. This is a valid approach under the assumptions of two-dimensionality, i.e. unidirectional waves. The length of the numerical domain $L_x= 40$ m with the number of nodes $N_x=1000$. The depth, d=2 m to match experiments and the number of *z* nodes, $N_z=10$, with σ values provided in Fig. 22. These values demonstrated converged results and are equivalent to many earlier studies (e.g. Li et al. (2023b); Draycott et al. (2022); Draycott and Li (2025); Tang et al. (2021); Moss et al. (2025)). The timestep Δt was set to 1/32 = 0.03125 s using a fourth-order Runge–Kutta time integration scheme which results in a Courant number between 1.46 to 2.17 depending on the celerity associated with $T_p$ (see Table 1).

As depicted in Fig. 1, a double relaxation zone (see, e.g. Perić et al. (2022)) was implemented, each 5 m in length (from *x* = −22.5 m to −12.5 m) for controlled wave generation and absorption, where a linear surface-elevation time series is defined at x=-17.5 m, i.e. between the relaxation zones (using Eq. 2). A damping pressure zone is applied for the final 5 m of the numerical domain. The relaxation-zone length ($L_{\textrm{relax}}$) was set to 5 m to ensure that $L_{\textrm{relax}}/L_p\ge 1$ for all cases, where $L_p$ is the wavelength associated with the peak frequency. $L_{\textrm{relax}}/L_p$ ranges from 2.2 to 1 for the sea states defined in Table 1. The relaxation zones are designed to minimise the generation of spurious free (error) waves which can be significant using wavemakers (Schäffer 1996), whilst also providing both wave generation and wave absorption capability. This choice is consistent with optimisation studies for relaxation-zone design (e.g. Perić et al. (2022)), which show that choosing the relaxation-zone thickness to be of the order of one wavelength ($L_{\mathrm {\textrm{relax}}}/L_p \ge 1$) is sufficient to keep numerical reflection coefficients small for engineering purposes.

Since the OW3D model does not allow overturning as it requires a single-valued free surface, wave breaking is not captured, but its dissipative effects are approximated using a breaking filter. In this implementation, this is applied when the vertical acceleration ($a_z$) is greater than 0.5 *g*, where *g* is the acceleration due to gravity. When activated, the filter acts locally by smoothing a short region around the exceedance location, following the OceanWave3D implementation described by Schløer (2013), in which a 10-point region is smoothed using a 3-point filter. In practice, the threshold value of $a_z/g$ is effectively used as a tuning parameter, and the value used here is in close agreement with earlier studies, e.g. Li et al. (2023b); Tang et al. (2021). As noted in Sect. 2, linear wave boundary conditions are defined in the form of a surface-elevation time series at the interface between the relaxation zones (*x* = −17.5 m).

### OpenFOAM

OpenFOAM is an open-source CFD solver with broad applicability and adaptability. In this study, we use a two-phase Large Eddy Simulation (LES) model to solve the incompressible LES-filtered Navier–Stokes equations (as in Li et al. (2023a)). Here, the multiphase flow solver olaFlow (Higuera 2015) is used within the OpenFOAM framework because it provides established wave generation and absorption methods for numerical wave tanks, including the hinged-flap wavemaker and enhanced wave absorption treatment used here (Higuera 2020). Although the present setup is two-dimensional, the LES closure is retained as a subgrid-scale model. We note that switching off the LES closure changes the effective dissipation and can therefore affect the wave field, particularly for the steepest and breaking cases. We, therefore, retain LES in all OpenFOAM simulations, including non-breaking cases, to provide a consistent, controlled subgrid dissipation across wave conditions. In this 2D (*x*, *z*) model, the flow is uniform in the transverse direction, so the LES model does not resolve inherently 3D turbulent structures, but it provides controlled dissipation of unresolved small-scale turbulence whilst allowing the larger-scale unsteady dynamics associated with both waves and wave-induced turbulence to be resolved. This choice is supported by a study comparing LES and RANS k-ϵ models indicating that the 2D LES approach gives better agreement with experiments for breaking waves (Zhang and Buldakov 2025). In order to capture the interface between the air and the water phase, in olaFlow, the volume-of-fluid (VoF) formulation with MULES-based interface compression is applied, where the volume fraction is governed by a transport equation. Our objective is not to reproduce the full three-dimensional turbulence cascade during breaking, but to compare phase-resolved surface elevation and the evolution of wave-induced kinematics between solvers for long-crested, nominally unidirectional sea states. In this context, the dominant breaking features in the (*x*, *z*) plane, including roller-type structures and associated bulk dissipation behaviour, can be assessed using the present configuration.

Time integration is conducted using the Crank–Nicolson scheme with a timestep of 0.001 s, coupled with a grid resolution of Δx=0.0156 m horizontally and $\Delta z=1/16 H_{m0}$ vertically (depending on the significant wave height (see Table 1). This provides between 144 and 320 points per peak wavelength across the range of cases. The very high temporal and spatial resolution is due to earlier tests where excessive numerical dissipation was noted (specifically with 0.002 s and 0.005 s timesteps, results presented in Fig. 18 for 0.005 s timesteps). From the output logs, the Courant number, calculated by OpenFOAM based on the local maximum velocity in the domain, remained below 0.2. Using the same celerity-based definition as OW3D (Sect. 2.3), the CFL numbers range between 1.19 and 1.78. The mesh used is depicted in Fig 21. Sensitivity tests for mesh and timestep are summarised in Appendix C, demonstrating that the chosen discretisation provides stable surface-elevation predictions at the target location.

As depicted in Fig. 1, the OpenFOAM model is configured to effectively be a 2D representation of the FloWave basin (single cell in the *y* direction), under the assumption of unidirectionality. To generate waves, a flap-type wavemaker with a hinge depth of 1.7 m is used, equivalent to FloWave, with motions imposed from linear wave theory according to Eqs. 4,5,6. To limit the size of the domain, and therefore computational time, an active wave absorption zone is implemented according to Higuera (2020), which is applicable for general water depth. Wave absorption is not applied at the moving hinged flap itself, which follows the prescribed kinematics only (Eq. 6). At the downstream boundary, wave absorption is implemented using the Enhanced Wave Absorption (EWA) method in olaFlow (Higuera 2020). In the present setup, absorption is applied through the EWA boundary treatment (without a relaxation zone or internal-field relaxation). The total domain length is, therefore, equivalent to the 25 m diameter of the circular FloWave basin.

## Results

The results are presented in this section, split into surface elevations (Sect. 3.1) including comparison to experiment, kinematics (Sect. 3.2), and dispersion and dissipation (Sect. 3.3).

### Surface elevations

#### Time and frequency domain

**Fig. 2:**
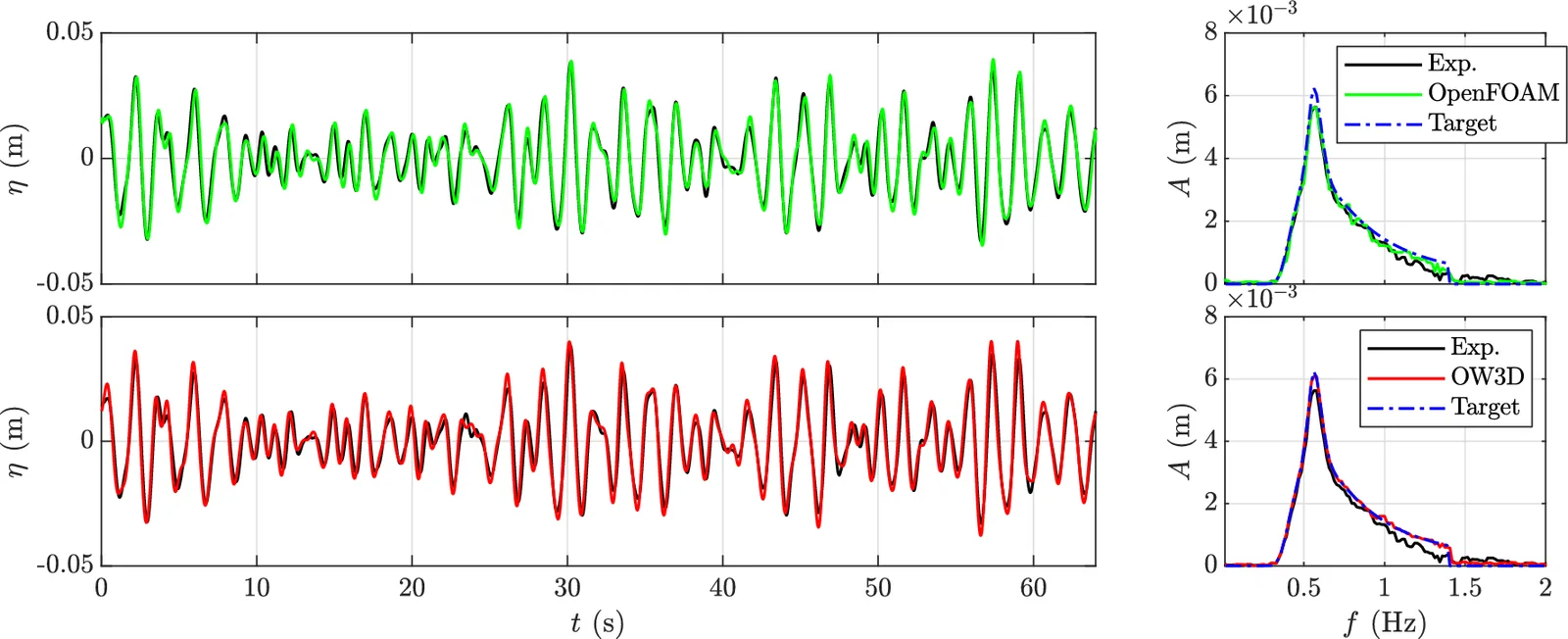
Time series and FFT comparisons at x=0 for case 3 (see Table 1)

**Fig. 3:**
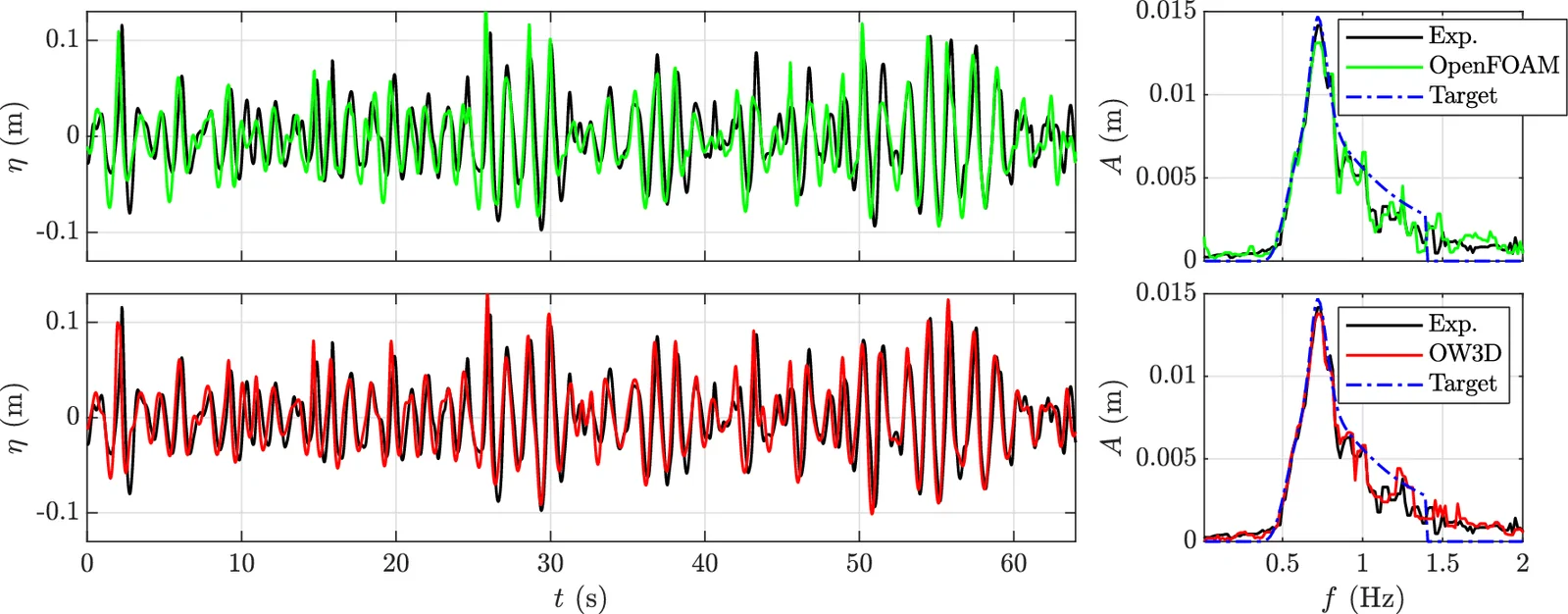
Time series and FFT comparisons at x=0 for case 10 (see Table 1)

At the target position of $\textit{x}=0$, the time series and spectra, computed using a Fast Fourier Transform (FFT), for 2 example cases are presented in Figs. 2 and 3 with all 11 cases presented in Figs. 25 and 26. For the example conditions, case 3 and 10 are shown as representative examples spanning the range of peak periods and steepness with case 10 being a breaking condition, illustrating both the more challenging short-period regime and a longer-period lower steepness case. Comparisons are made to the ‘target’ spectrum which denotes the discretised linear spectrum used to prescribe the input wave components (Eq. 1). Because the input is defined on discrete frequency bins and spectra are computed over the repeat period $T_r$, the resulting FFT resolves the prescribed bins directly and minimises spectral leakage.

It is evident that in general there is good agreement between the experiments and model outputs for both models for all cases. Figure. 2 shows case 3 with a low wave steepness (see Table 1). For this condition, both OpenFOAM and OW3D agree with the experimental data based on the time histories. In the frequency domain, both models accurately capture the spectrum. OW3D slightly overestimates the amplitude at the peak frequency and at high frequency relative to the measurements, yet it is closer to the target spectrum. This could be related to the high resolution of OpenFOAM better resolving the smaller, high-frequency waves. However, given the better match to the target spectrum from OW3D, it is likely that the difference is in large part due to both FloWave and OpenFOAM having effectively the same approximate linear wavemaker transfer functions applied.

Figure 3 shows case 10 with a large wave steepness. For this case, OpenFOAM shows further deviation from the experiment, with notably larger crest amplitudes present in the time series. On the other hand, OW3D follows the experimental time series more closely. In the frequency domain, OW3D exhibits a slightly better match with the experimental data in the peak region, whilst the OpenFOAM results underestimate the amplitude spectrum slightly, possibly due to excess dissipation for this lower $T_p$ breaking condition (not observed for case 3 with higher $T_p$). Additionally, wave breaking, which has been observed in the experiments for this wave condition, will increase the physical dissipation. Wave breaking is accompanied by the generation of vortices and turbulence along with air entrainment which transfers energy from the wave scales to smaller scales which are ultimately dissipated as heat. In the present experiments, these turbulent processes contribute to the enhanced dissipation of the steeper breaking sea states (cases 7 and 10). OW3D represents their net effect only through the local smoothing scheme, whilst OpenFOAM resolves the larger-scale turbulent structures within the LES-VoF framework. As these models both capture these events very differently, increased deviation is expected between the models. For both models, and the experiments, there is larger deviation from the target linear incident spectrum which is explained by nonlinear wave–wave interactions and wave breaking processes. Overall, for the steeper and shorter-period sea states, these deviations should, therefore, be viewed as arising from a combination of physical processes (breaking, breaking-induced turbulence and nonlinear energy transfers) along with model-related limitations (e.g. grid-dependent numerical dissipation in OpenFOAM and the parametrised breaking dissipation in OW3D), rather than from purely numerical error in either model.

**Fig. 4:**
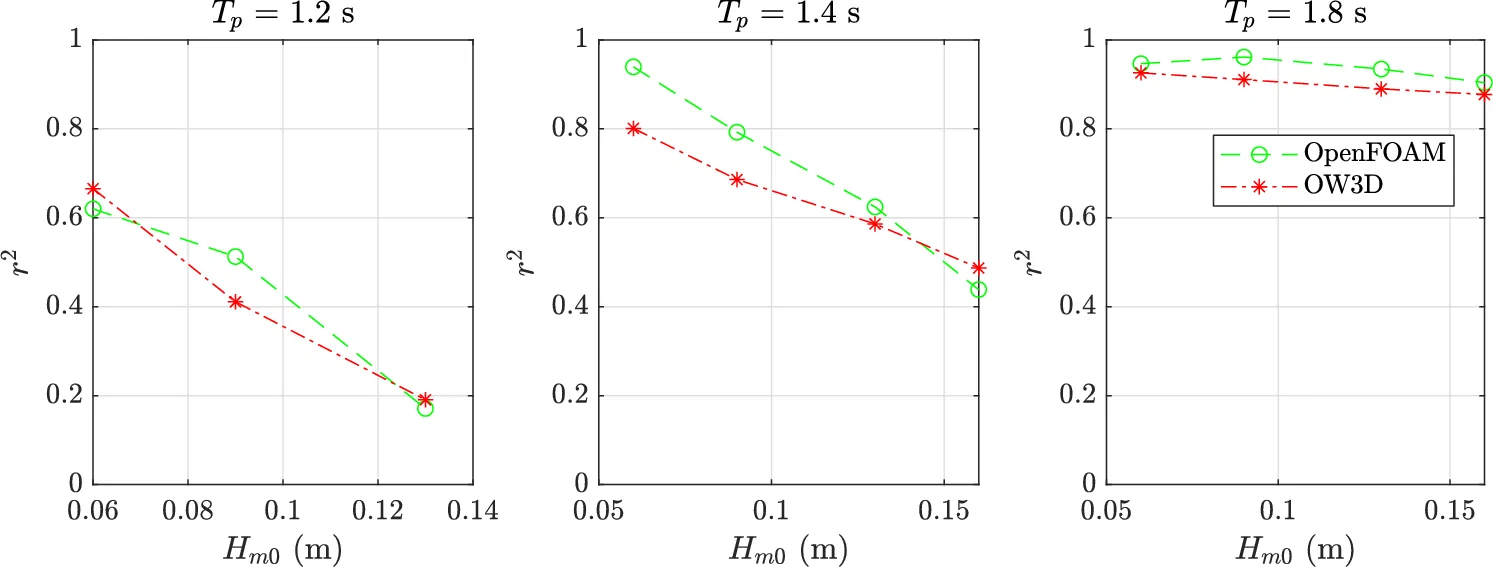
Raw $r^2$ values between time-domain surface-elevation numerical model outputs and experiments at x= 0 m. Shown for cases 1 to 11 (see Table 1) as a function of $H_{m0}$ for different values of $T_p$

To assess the performance in all cases, coefficients of determination ($r^2$) between measurements and model outputs at x=0 m are presented in Fig. 4 for both OpenFOAM and OW3D results. Over all cases, it is shown that the raw OpenFOAM data generally achieves higher $r^2$ values than OW3D. As the wave height increases and the wave period decreases, the accuracy of both results decreases rapidly, especially for shorter wavelengths with $T_p$=1.2 s, where the *r*^2^ is only around 0.2 for both models. The reasons can be understood by examining the differences in the time-series results compared to experimental data, where a significant phase shift in the time domain is evident between them. This time shift increases with the steepness of the waves and the associated nonlinear effects, and is expected to be a result of observed phase mismatches in the generation of high-frequency waves in a circular tank, along with different wave generation mechanisms. These phase mismatches for high-frequency waves were also observed in Draycott et al. (2022). Although the precise reasons remain unclear, they are expected to be associated with the use of linear wavemaker theory for short-wavelength cases, where the phase differences between adjacent paddles around the circumference are large.

**Fig. 5:**
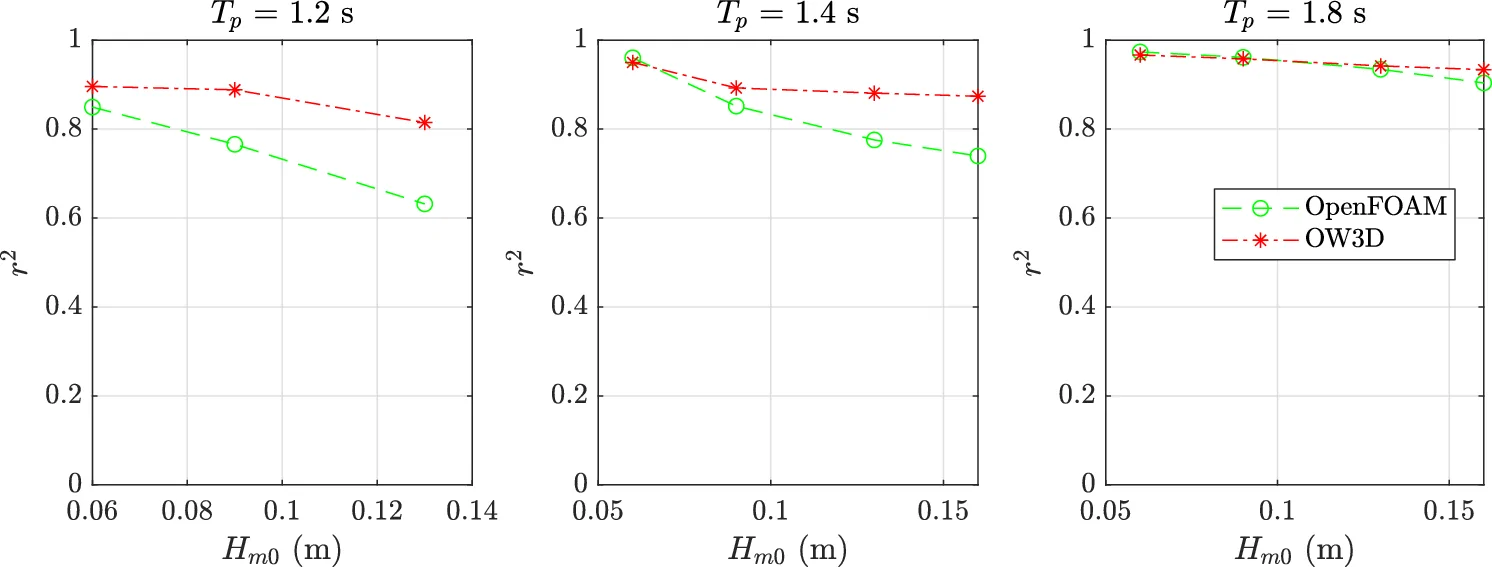
$r^2$ values between time-domain surface-elevation numerical model outputs and experiments at x= 0 m after applying a single time shift based on maximising cross-correlation. Shown for cases 1 to 11 as a function of $H_{m0}$ for different values of $T_p$

To crudely account for the phase offset, we perform cross-correlation analysis and remove the associated time lag to maximise cross-correlation between measurements and model outputs. This will give the best ‘bulk’ phase offset which can be applied to align the time series, and enables spatial comparisons (Figs. 7 and 8). These results are presented in Fig. 5, with corresponding lags presented in Appendix Appendix E. It is noticeable that the adjusted *r*^2^ values show a clear and substantial improvement across various conditions. For instance, in the case with a peak period of 1.2s, the minimum *r*^2^ value increases from 0.2 to over 0.6. Notably, after these adjustments, the OW3D results exhibit higher *r*^2^ than OpenFOAM, aligning more closely with the experimental data. The reason behind the larger performance increase when accounting for a fixed time lag for OW3D is likely due to the larger differences in wavemaking approach and effective distances between wave generation and target position, which are somewhat accounted for with a fixed time lag.

**Fig. 6:**
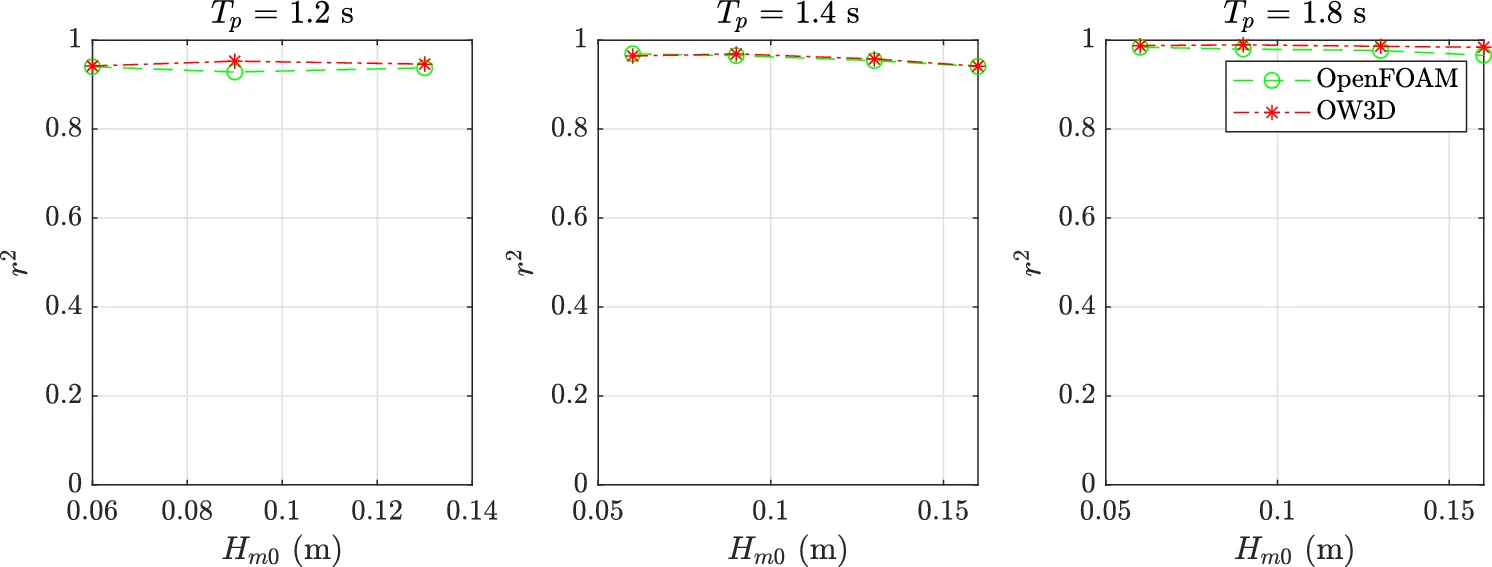
$r^2$ values between numerical model outputs and experiments for FFT surface elevation amplitudes (0.1 → 2.0 Hz) at x= 0 m. Shown for cases 1 to 11 as a function of $H_{m0}$ for different values of $T_p$

Coefficients of determination between experimentally measured and model output surface-elevation FFTs are presented in Fig. 6. The results indicate that both OpenFOAM and OW3D exhibit extremely high *r*^2^ (close to 1) for all cases. There is no significant difference between the two models across the range of significant wave heights or peak periods, indicating that both models accurately capture the surface-elevation amplitudes in these scenarios, regardless of the wave height or period.

#### Spatial comparisons

**Fig. 7:**
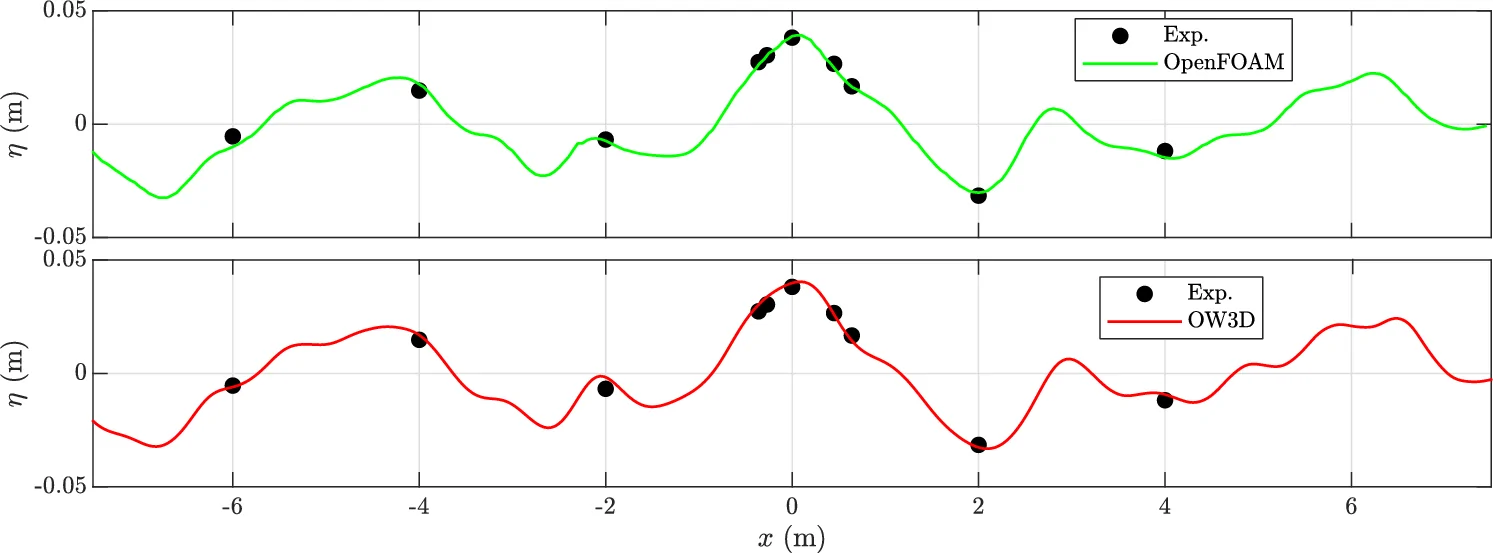
Comparisons of spatial surface elevation at the time of maximum in experiments and equivalent time in numerical model (after applying time shift). Shown for case 3 (see Table 1)

**Fig. 8:**
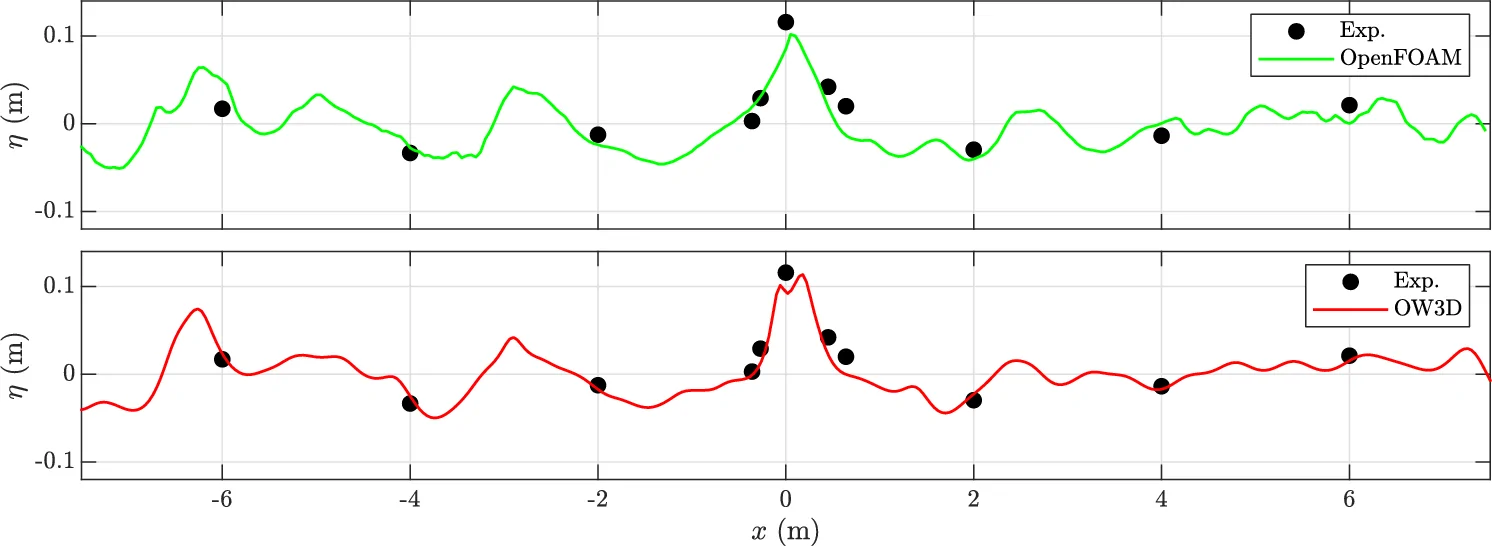
Comparisons of spatial surface elevation at the time of maximum in experiments and equivalent time in numerical model (after applying time shift). Shown for case 10 (see Table 1)

Figures 7 and 8 show comparisons of spatial surface elevation from experiments and numerical models for cases 3 and 10, respectively, with time lags removed to maximise cross-correlation as described in Sect. 3.1.1. Equivalent results for all cases are presented in Figs.27 and 28. For case 3 presented in Fig. 7, both OpenFOAM and OW3D capture the wave profiles quite well, with both models closely following the experimental data under lower wave steepness conditions. For case 10 (Fig. 8), the performance of both models deteriorates and there are more substantial deviations from the experimental data in both the peaks and troughs of the measured waves, likely owed to wave breaking effects and challenges with modelling higher wavenumber components. Overall a reasonably good agreement is found between the spatial elevations for both numerical models.

**Fig. 9:**
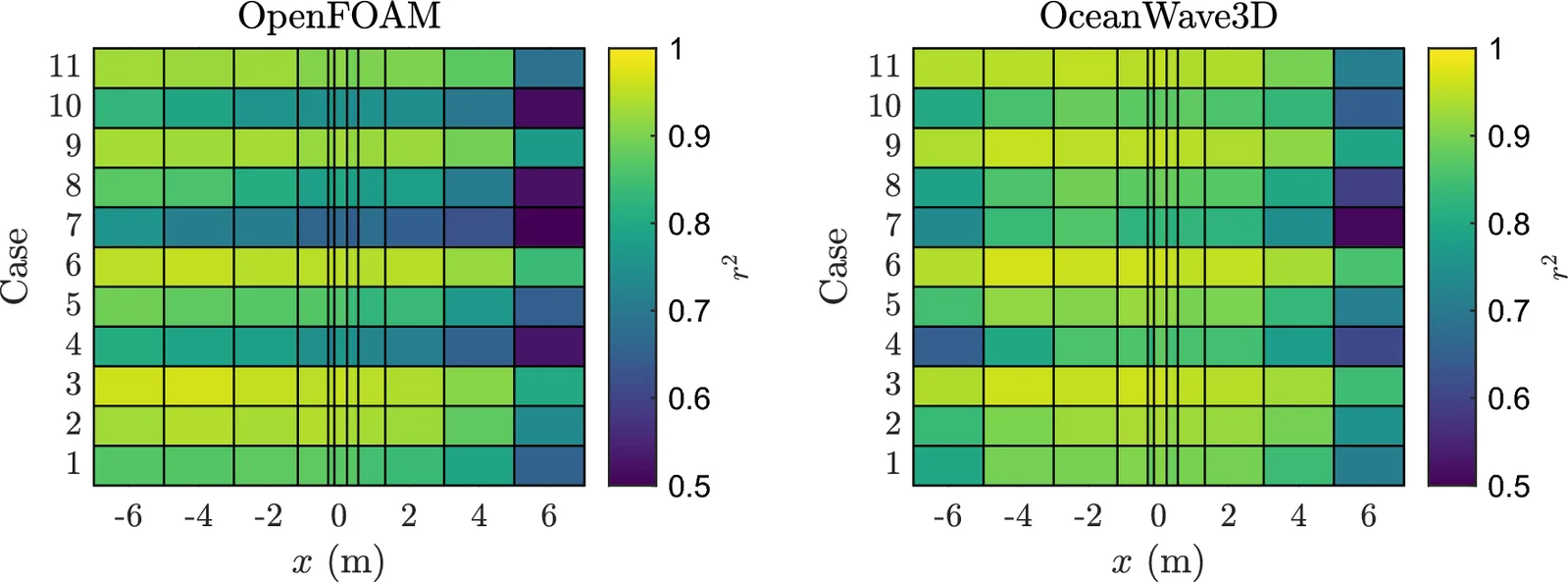
Spatial comparisons of $r^2$ for OpenFOAM and OceanWave3D relative to the experimental surface elevation with reflections removed. A single bulk phase shift is applied per case and used consistently across all gauges. Results are shown for cases 1–11 (see Table 1) and for all experimental wave-gauge positions (see Table 2)

To further assess comparisons spatially, we compute the coefficients of determination, $r^2$, for both OceanWave3D and OpenFOAM at each experimental wave-gauge position. A single bulk phase shift is first applied for each case, and this time shift is then used consistently across all gauges (as in Fig. 5). Results are presented in Fig. 9. In general, similar agreement to that reported at x=0 is found over a large area surrounding x=0, particularly for negative *x*. However, agreement is reduced for large positive *x*, which we attribute to the circular focusing of reflections in FloWave observed near this region (see Draycott (2017)). Reflection estimates taken near the tank centre are only strictly accurate local to that region, and therefore residual reflection effects and imperfect decomposition can become more apparent further away. Consistent with Fig. 5, OceanWave3D shows higher agreement with the experimental measurements across most cases and spatial locations. Both models agree more strongly with the experimental data for larger $T_p$ conditions (e.g. cases 3, 6, and 9, see Table 1) and lower steepness. Cases 4, 7, and 10 show larger discrepancies due to their higher steepness and shorter wavelengths and, for cases 7 and 10, the presence of breaking.

#### Crest distributions

**Fig. 10:**
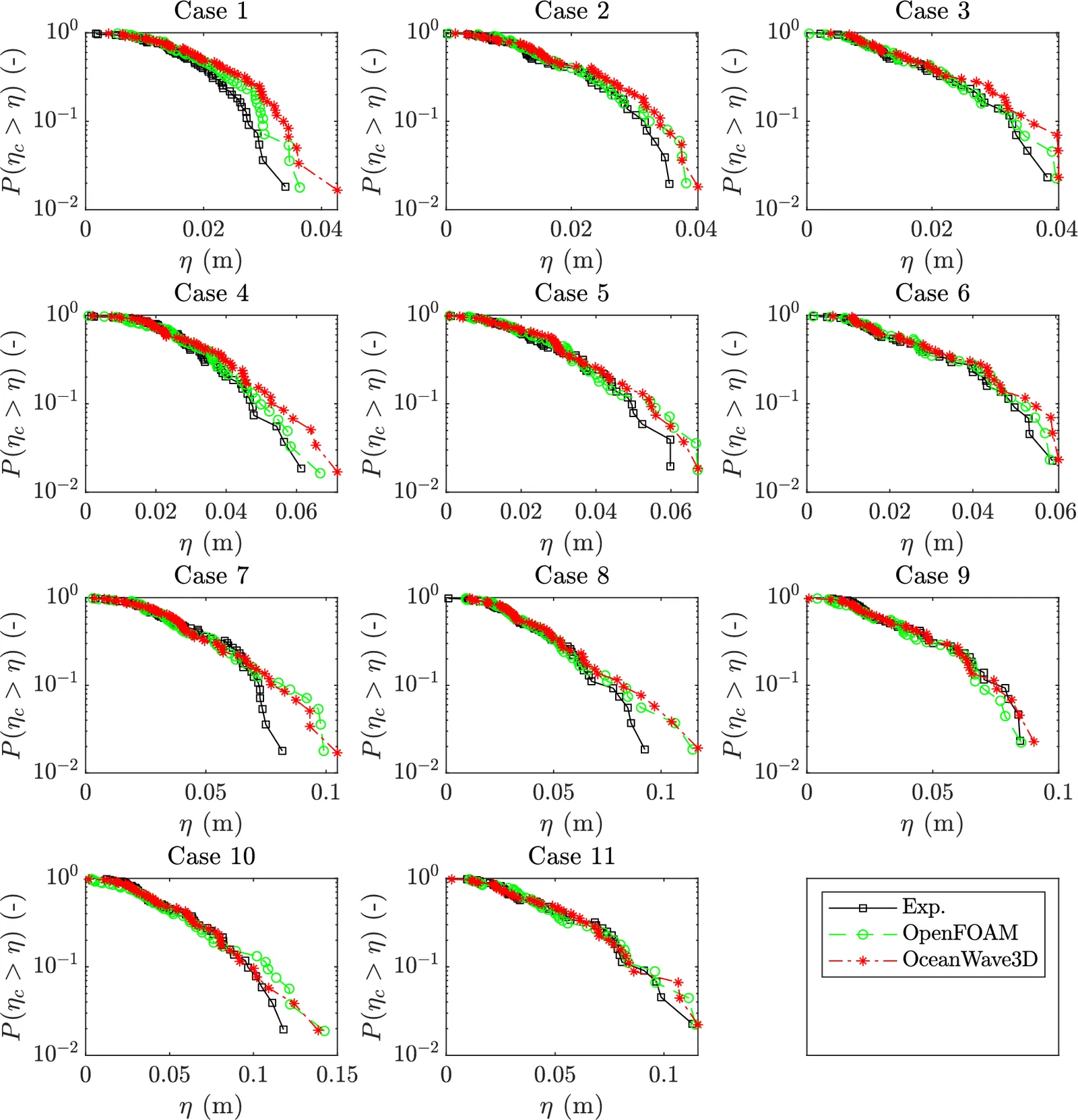
Comparisons of crest amplitude distributions for cases 1 to 11 at *x* = 0 m. The $H_{m0}$ and $T_p$ values these correspond to are presented in Table 1

For all 11 cases, wave crest amplitudes are computed and empirical crest distributions are presented in Fig. 10. These short-run sea states clearly do not have converged or representative statistics, but can be used as a mechanism to compare between like-for-like cases. In general, it is apparent that for the longer wavelength and lower steepness cases, there is the best agreement between the two models and between models and experiment, with excellent agreement found for all cases with $T_p$ = 1.8 s (cases 3, 6, 9 in the third column of Fig. 10) and the majority of $T_p$ = 1.4 s (cases 2, 5, 8, 11 in the second column of Fig. 10). For cases where there is larger disagreement between experiment and model outputs, e.g. case 7 ($T_p = 1.2$ s), there is still strong agreement between the two models. One aspect of uncertainty in the comparisons here is the effect of reflections which have been removed under linear assumptions from the experiments, yet should be negligible in the numerical model results. Reflections are known to be larger in FloWave for lower period conditions (Draycott 2017), which is affecting the ability to fairly compare.

### Kinematics

#### Comparisons

**Fig. 11:**
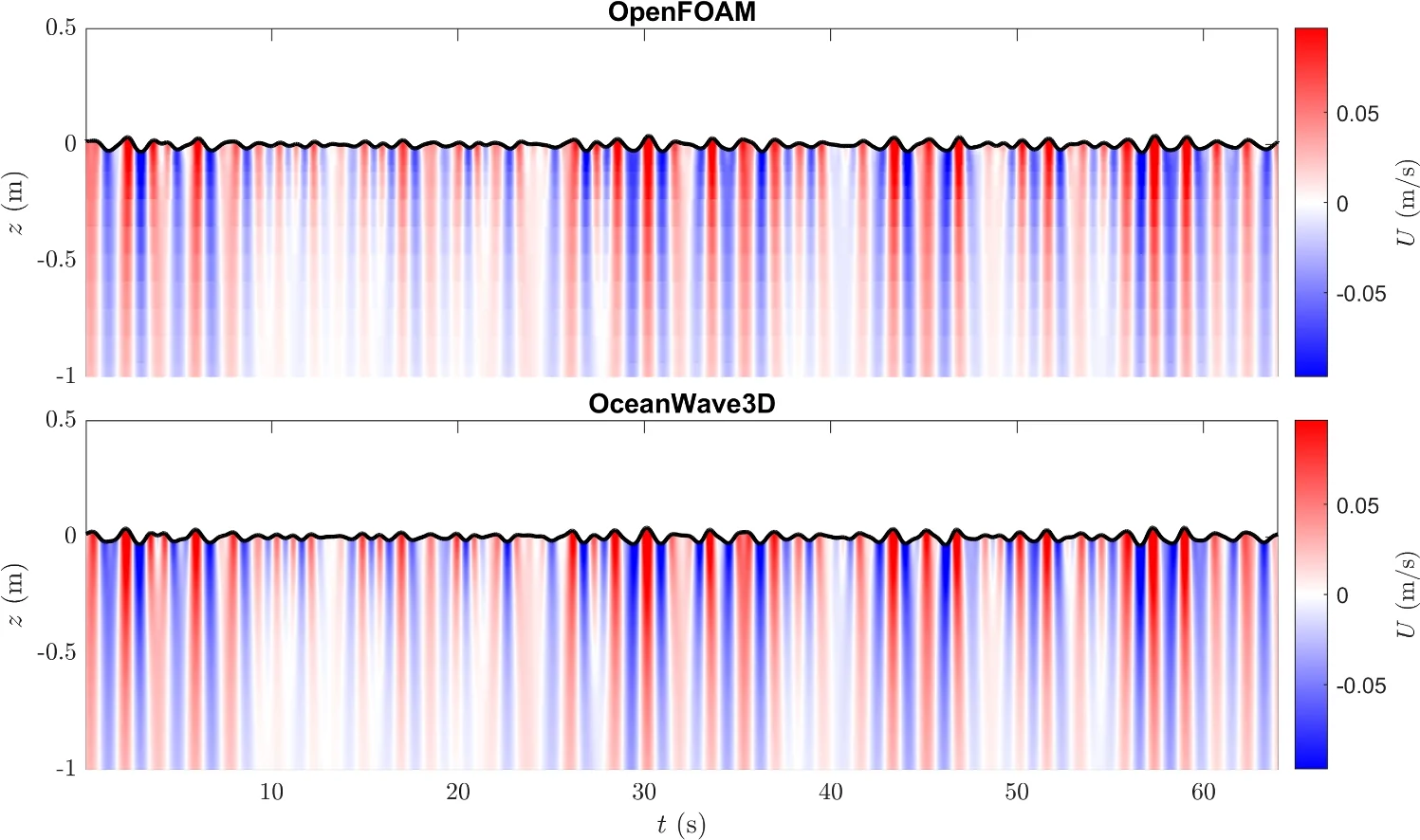
Comparisons of time series of horizontal velocity profile *U*(*z*, *t*) at *x* = 0 m for case 3 (see Table 1)

**Fig. 12:**
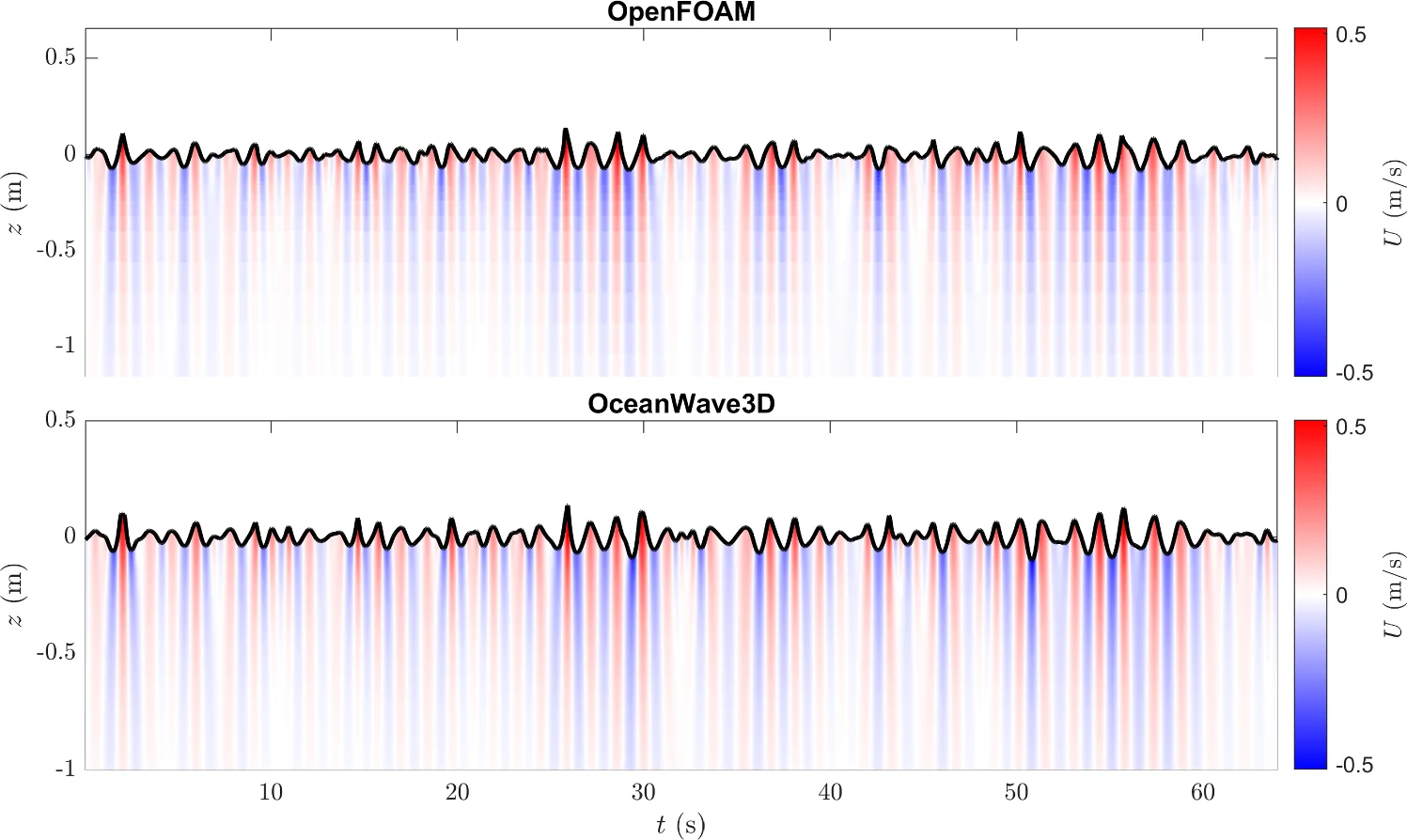
Comparisons of time series of horizontal velocity profile *U*(*z*, *t*) at *x* = 0 m for case 10 (see Table 1)

**Fig. 13:**
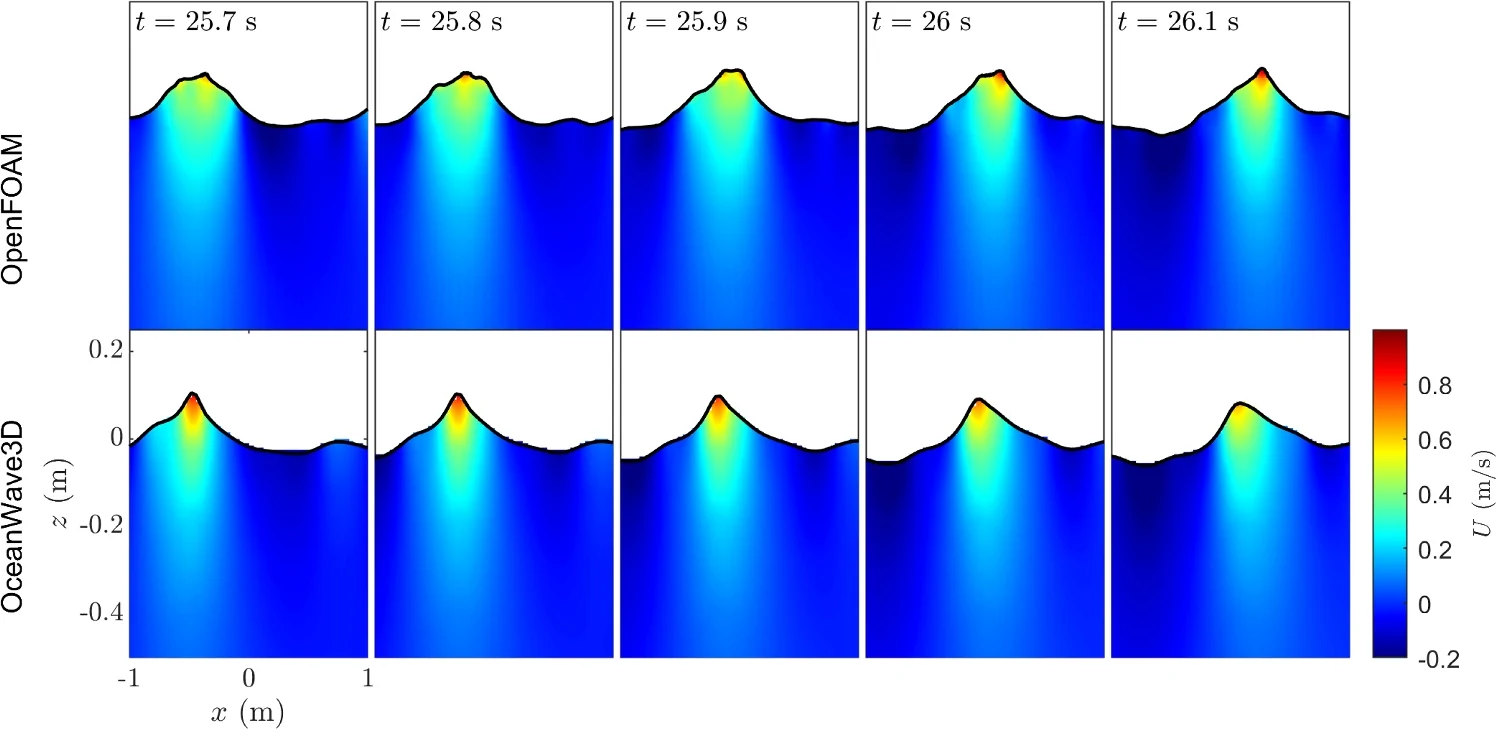
Evolution of the spatial wave profile and sub-surface horizontal velocity during wave breaking for case 7 (see Table 1), after applying a time shift to enable fair comparisons

**Fig. 14:**
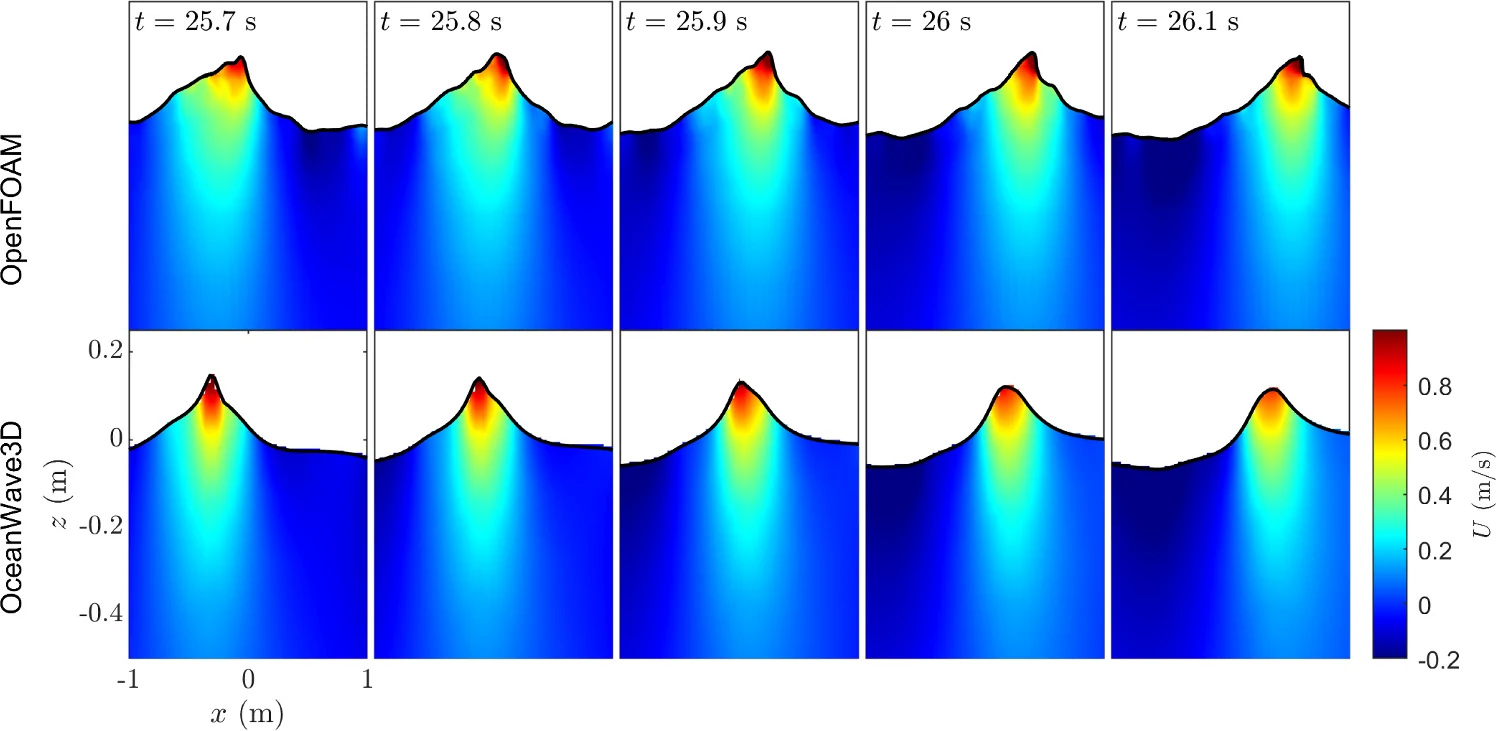
Evolution of the spatial wave profile and sub-surface horizontal velocity during wave breaking for case 10 (see Table 1), after applying a time shift to enable fair comparisons

Comparisons of the horizontal velocity as a function of *z* and *t* (at *x* = 0) are presented in Figs. 11 and 12, for cases 3 and 10, respectively. There is clearly excellent agreement in the wave group structure and associated sub-surface kinematics. At a single point in *x*, the presence of wave breaking is not easy to identify. As such, spatial comparisons are made for the steepest wave cases where wave breaking was observed: cases 7 and 10. These are shown for instances where breaking is observed in OpenFOAM over a 0.4 s period for case 7 in Fig. 13 and case 10 in Fig. 14, where time lags have been removed to facilitate comparisons. The difference between the models is now much clearer, where in OpenFOAM, the presence of mild spilling breaking and very high crest-front velocities are observed along with the associated generation of high-wavenumber surface-elevation components. At the same time steps in OW3D, there is no evidence of wave breaking or local smoothing being applied.

#### Statistics

**Fig. 15:**
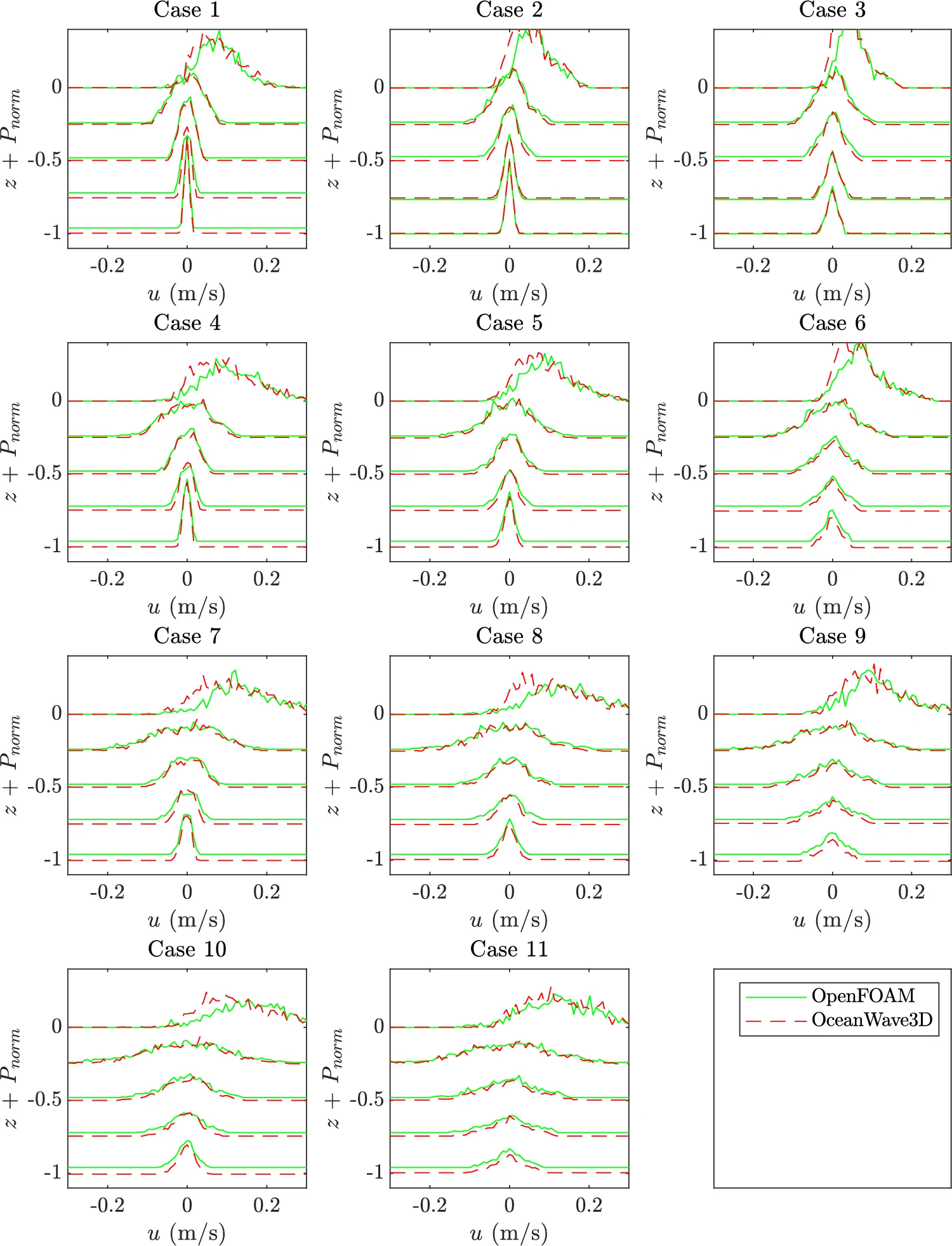
Normalised probability distributions $P_{\textrm{norm}}$ (histograms) of horizontal velocity at different water depths. The $H_{m0}$ and $T_p$ values these correspond to are presented in Table 1

To further assess the differences in kinematics between OpenFOAM and OW3D, distributions of the horizontal velocity at different depths are presented in Fig. 15 for all cases. These are calculated at *z* = 0 m (i.e. the still water surface) and at *z* = −0.25 m, −0.5 m, −0.75 m and -1 m from the surface. The probability distributions have been normalised ($P_{\textrm{norm}}$) in order to fit on the *y* scale. At depths below the near-surface region, the horizontal-velocity distributions predicted by the two models agree very closely and are well approximated by normal distributions, consistent with linear wave theory. Closer to the surface, there are greater deviations from normal distributions, and there is greater asymmetry between positive and negative velocities as would be introduced by bound waves due to weak nonlinearity. For all depths other than z=0, there is still excellent agreement between the two models. At z=0, there are larger discrepancies observed. First, as expected, there are only positive values of the horizontal velocity observed due to only capturing the wave crests. Additionally, there is a noticeable shift in the distribution to lower velocities for the OW3D model outputs, which are attributed to the differences in treatment of the free surface and the much lower grid resolution in the vertical direction for OW3D compared to OpenFOAM.

### Dispersion and dissipation

**Fig. 16:**
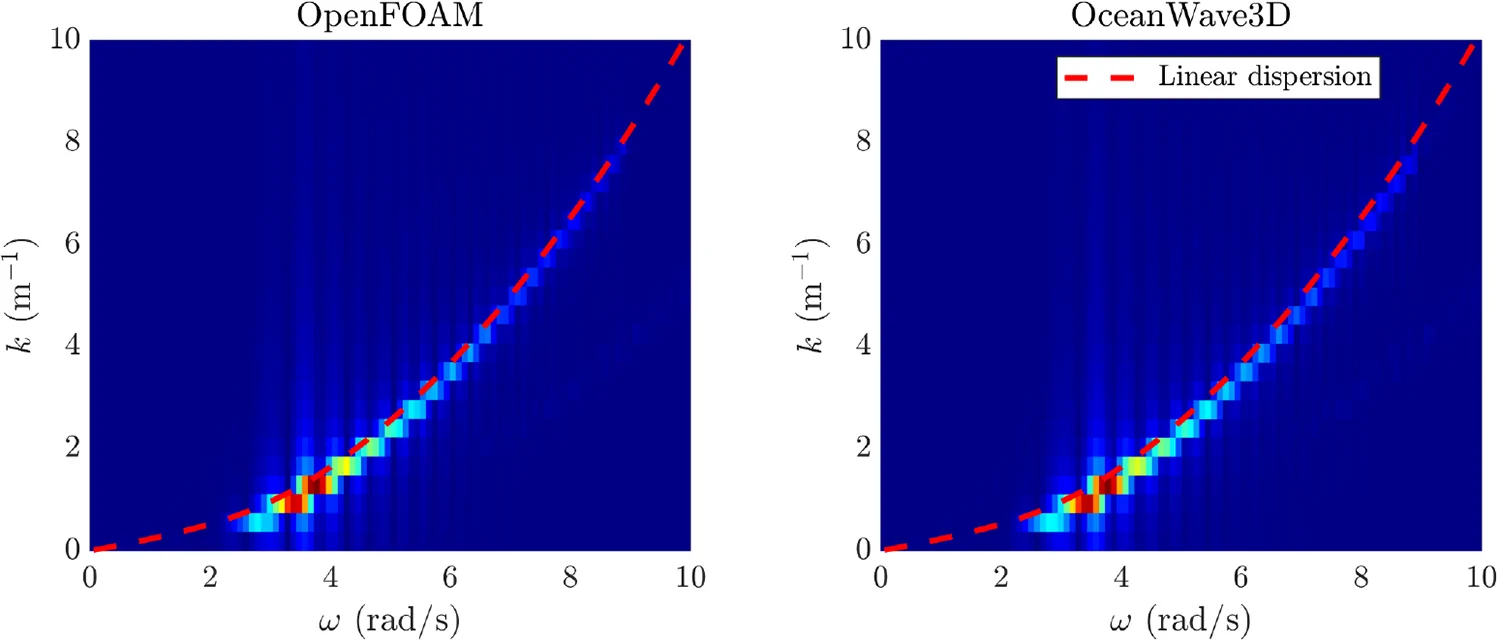
2D FFTs for case 3 (see Table 1) compared to the linear dispersion relation (red dashed line)

**Fig. 17:**
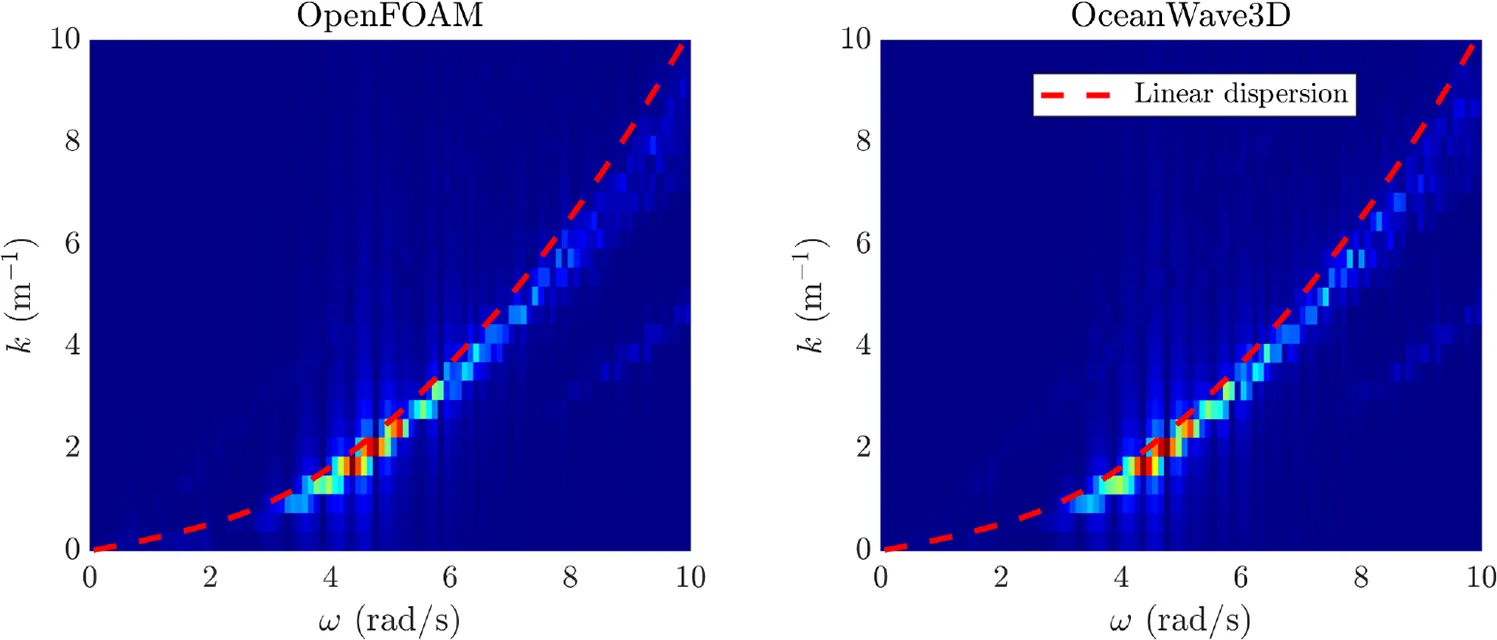
2D FFTs for case 10 (see Table 1) compared to the linear dispersion relation (red dashed line)

This section assesses differences in dispersion and dissipation. To assess wave dispersion characteristics, we take 2D (*x*, *t*) Fast Fourier Transforms (FFTs) of the surface elevation. As such we cannot present experimental results due to insufficient number of gauges. Results are presented for both OpenFOAM and OW3D in Figs. 16 and 17 for cases 3 and 10, respectively. For case 3 in Fig. 16, both the OpenFOAM and OW3D results appear to be near identical to each other and agree very well with the linear dispersion relation presented in the red dashed line, even for high-frequency waves. For case 10 presented in Fig. 17, there is overall very similar agreement in the models; however, there is a slight increase in the amplitudes of high-frequency/wavenumber components relative to OpenFOAM which is attributed to dissipation due to wave breaking. For this steeper wave condition, both OpenFOAM and OceanWave3D deviate from the linear dispersion relation due to nonlinear dispersive effects.

**Fig. 18:**
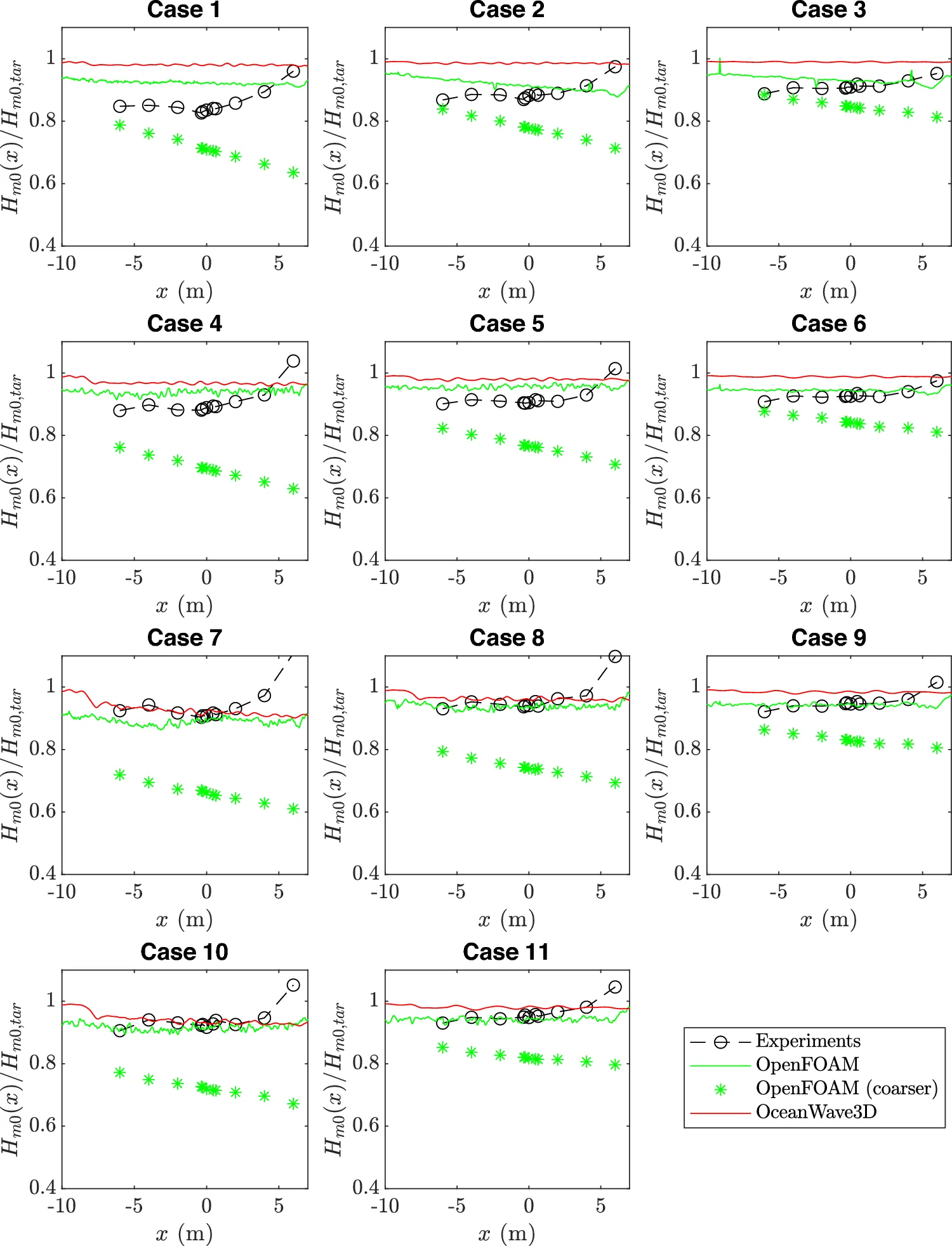
Spatial variation of $H_{m0}$ for each sea state, showing experiments, OceanWave3D and OpenFOAM. The $H_{m0}$ and $T_p$ values these correspond to are presented in Table 1. Also shown is the dissipation for earlier runs using OpenFOAM with coarser resolution

The dissipation analysis is presented in Fig. 18, through assessing the spatial variation of $H_{m0}/H_{m0,\textrm{tar}}$ where $H_{m0,\textrm{tar}}$ is the target significant wave height as defined in Table 1. For OW3D, it is evident that there is an excellent match between the target and observed $H_{m0}$ with minimal dissipation (i.e. a flat line around 1), other than for cases 7 and 10 where dissipation is observed which is attributed to wave breaking. Experimental measurements and OpenFOAM tend to under-produce the target wave conditions which is likely due to errors in the linear flap-type wavemaker transfer functions which are applied both numerically in OpenFOAM and experimentally in FloWave. Some numerical dissipation is observed in the OpenFOAM simulations; however, this is relatively small owed to the very high spatial and temporal resolutions used for the modelling. There is evidence of dissipation in experiments; however, this is complicated by errors in the reflection analysis approach far away from x=0. As described in Draycott (2017), the reflected waves in FloWave are spatially variable and curved due to the circular geometry (although locally approximated as opposing the incident waves), and as such, reflection analysis is only strictly accurate local to the reflection measurement, i.e. around gauges 4–8. The gauges far away from this location, particularly around x=+6 m are observing a very different reflected wave field, and as such, reflections are not effectively removed. This is one of the reasons behind focusing close to x=0 for comparisons throughout the paper. Also shown in Fig. 18 is the dissipation for earlier coarser runs with OpenFOAM, highlighting the potential for very excessive dissipation if timesteps are too large (0.005 s in this instance).

## Model strengths and limitations

The two numerical approaches considered here are representative of two widely used classes of wave model. OceanWave3D (OW3D) is an FNPF solver which assumes inviscid, irrotational flow with a single-valued free surface. The governing Laplace equation is discretised using a sixth-order finite-difference scheme in space and a fourth-order Runge–Kutta scheme in time (Ducrozet et al. 2010). Nonlinearity in the free-surface boundary conditions is treated exactly, but overturning cannot be resolved due to single-valued free surface requirement, and nor can air-entrainment. Wave breaking is represented via a local smoothing function which enforces dissipation based on a vertical-acceleration threshold.

In contrast, the OpenFOAM setup used here is a two-phase VoF Navier–Stokes solver with an LES turbulence closure as implemented in olaFlow (Higuera 2015). Spatial discretisation is formally second-order, and the Crank–Nicolson time integration is also second-order accurate. This configuration is, therefore, more general in terms of physics (viscous flow, vorticity, turbulence, air-water mixing, overturning free surface), but for a given grid and timestep, it is less numerically accurate than the high-order FNPF formulation. In practice, accurate results require finer meshes and smaller time steps, leading to substantially higher computational cost.

It is difficult to fairly compare the run times of the models as OW3D was run on a desktop PC, and OpenFOAM was run on a CPU cluster. Nevertheless, it is worth pointing out the large differences here. For OceanWave3D, a typical run required $\mathcal {O}(100~\textrm{s})$ wall-clock time on a single CPU core to simulate 100 s of physical time, giving an approximate cost of $C \approx 1/3600 \approx 2.8\times 10^{-4}$ core-hours per simulated second. For OpenFOAM, a typical run required $\mathcal {O}(40~\textrm{h})$ wall-clock time on 8 CPU cores to simulate 100 s of physical time, giving $C \approx (8\times 40)/100 \approx 3.2$ core-hours per simulated second. Based on these representative values, OpenFOAM is approximately $3.2/(2.8\times 10^{-4}) \approx 1.1\times 10^{4}$ times more expensive than OceanWave3D excluding post-processing. We note that costs vary between cases, particularly for OpenFOAM, where steeper and near-breaking/breaking conditions can increase solver iterations.

In the present study, both models are configured as effectively 2D numerical wave tanks. Whilst both codes can be run in three dimensions, the computational cost of OpenFOAM for long irregular realisations in 3D is generally prohibitive, and even FNPF models become expensive for large 3D domains. In the OpenFOAM setup, the LES closure is, therefore, applied in a 2D (*x*, *z*) domain, implying that the flow is uniform in the transverse direction and that inherently three-dimensional turbulent structures (varying in *y*) cannot be represented. Turbulent stresses are only accounted for in an effective, subgrid sense, without resolving spanwise-varying vortical structures. This is appropriate for long-crested, approximately two-dimensional wave fields and for turbulent structures that are effectively uniform in the transverse direction, but it cannot capture spanwise-localised features resulting from wave breaking. Neither approach achieves the accuracy of direct numerical simulation (DNS) of turbulence: OW3D neglects viscous and turbulent effects entirely, whereas the OpenFOAM setup relies on a subgrid-scale model whose accuracy depends on local grid spacing and model assumptions.

From a practical modelling perspective, OW3D performs particularly well for broadband and nonlinear sea states, but is best applied to predominantly non-breaking conditions. It reproduces the target spectrum with no numerical dissipation (see Fig. 18) and captures statistics of surface elevation and kinematics with high accuracy, whilst remaining computationally efficient. The key limitations are: (i) the inability to represent overturning crests, plunging or strong spilling breaking, and the associated air entrainment and turbulence; (ii) the omission of viscous phenomena. For applications focused on local impact loads from breaking wave impacts or detailed turbulence, an FNPF model is therefore not appropriate in isolation.

The OpenFOAM VoF–LES approach offers a more detailed and complete flow description at the cost of lower formal order of accuracy and much higher computational effort. In the present simulations, it is able to reproduce spilling breaking events, with locally high crest-front velocities and enhanced high-wavenumber content in both the free surface and the sub-surface velocity field (see Figs. 13 and 14). The same framework can be extended to include fixed or floating offshore systems with arbitrary geometry, providing pressure fields, loading, and detailed flow features around the body. The solutions are, however, quite sensitive to mesh resolution, time-stepping and numerical schemes, with insufficient resolution leading to excessive numerical dissipation (illustrated in Fig. 18), and the LES and VoF formulations still only approximate the smallest-scale turbulence and air entrainment.

Relative to other solvers, OW3D can be broadly seen as representative of other FNPF codes such as REEF3D-FNPF and higher-order spectral (HOS) numerical wave tanks (e.g. HOS-NWT, HOS-Ocean). Similarly, the OpenFOAM configuration used here is broadly representative of incompressible VoF Navier–Stokes solvers used in coastal and offshore engineering, including commercial codes such as STAR-CCM+ and Ansys Fluent/CFX. These solvers are based on equivalent governing equations and treatment of the free-surface, and the trade-offs between physical generality, second-order numerical accuracy and high computational cost are common to these types of numerical tools.

## Conclusion

This study has presented a like-for-like comparison between a fully nonlinear potential flow model (OceanWave3D) and a two-phase VoF CFD model (OpenFOAM) for the simulation of 11 well-defined irregular JONSWAP sea states, including cases close to limiting steepness. Both numerical wave tanks were driven by equivalent boundary conditions as the FloWave wave tank, and comparisons were made for surface elevations, kinematics, dispersion and dissipation.

At the target position (x=0 m), both models reproduce the measured surface-elevation time series and spectra very well across the range of sea states, once small bulk phase lags have been removed. Coefficients of determination for the spectra are close to unity for all cases, and crest statistics and subsurface velocity distributions show good agreement between models and experiment. OW3D reproduces the target spectrum with very little numerical dissipation and closely matches the crest statistics for the less steep conditions, whilst OpenFOAM tends to slightly under-predict the target energy level, consistent with the modest numerical dissipation evident in the spatial variation of significant wave height.

**Fig. 19:**
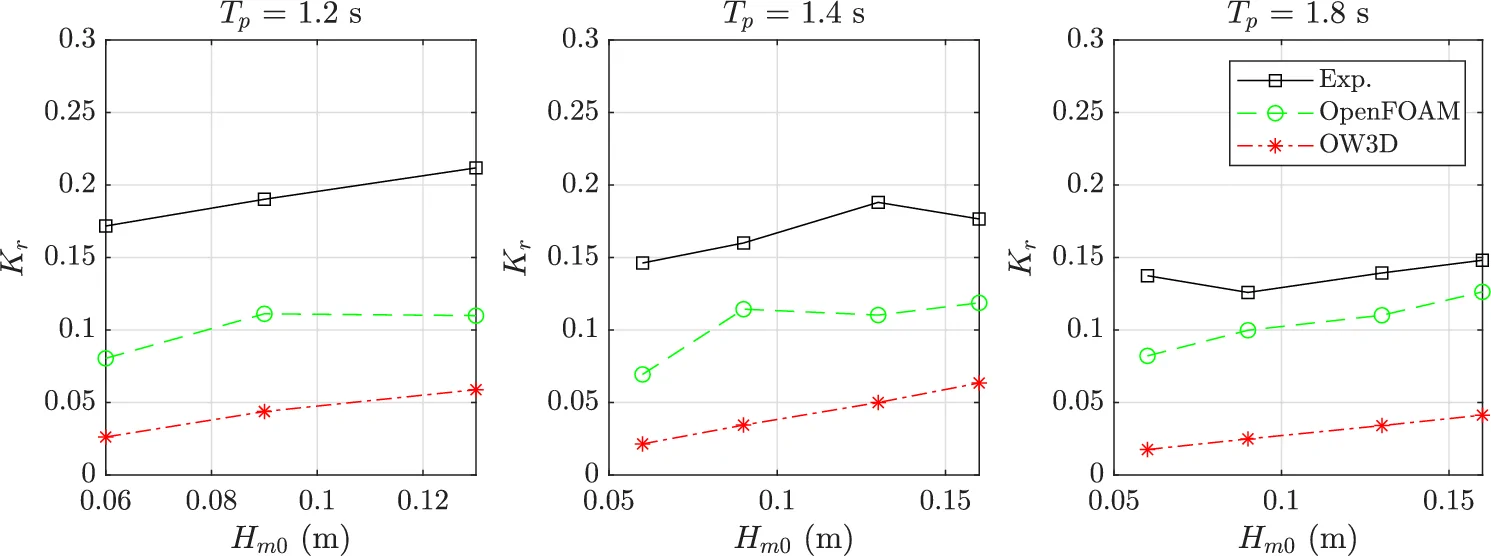
Total integrated values of the reflection coefficient $K_r$ for experiments, OpenFOAM and OW3D. Estimates are made near to the target position x=0 m and using consistent ‘gauge’ positions as experiments (gauges 4–8 in Table 2). Estimates are shown for cases 1 to 11 (see Table 1) as a function of $H_{m0}$ for different values of $T_p$

**Fig. 20:**
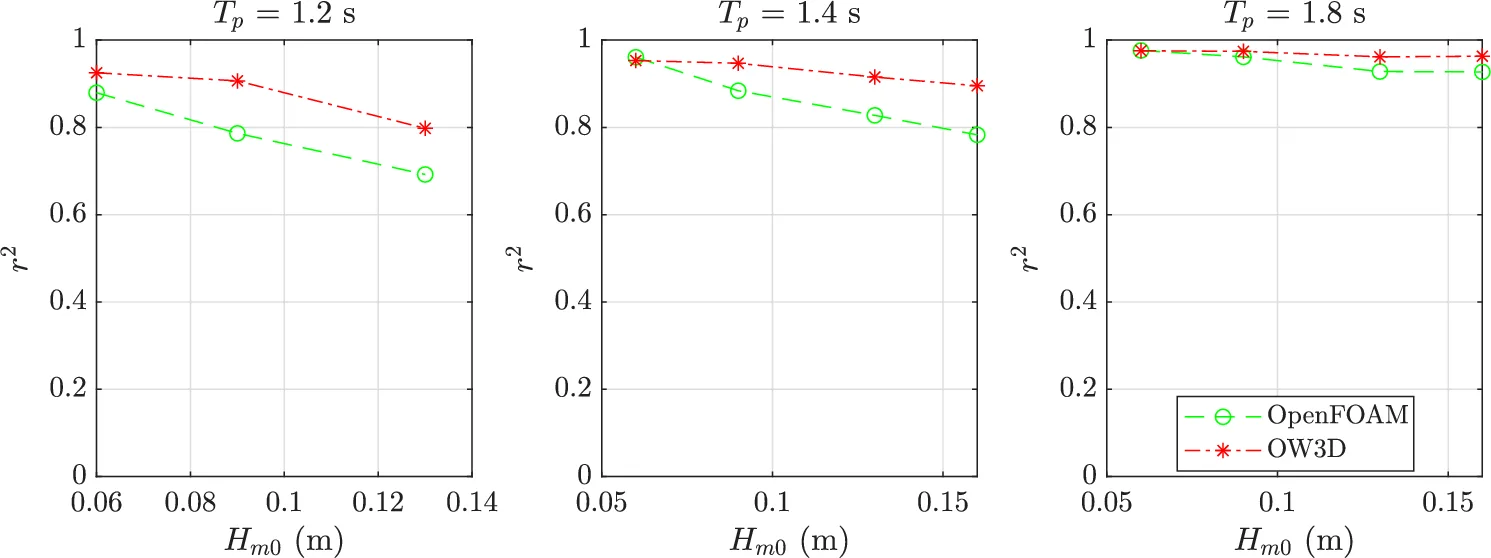
$r^2$ values between time-domain surface-elevation numerical model outputs and experiments at x= 0 m after removing reflections from all measurements and applying a single time shift based on maximising cross-correlation. Shown for cases 1 to 11 as a function of $H_{m0}$ for different values of $T_p$

For the steeper and shorter-period sea states, larger deviations from the target linear spectrum are observed in both experiments and models, and the OW3D and OpenFOAM time series show correspondingly larger departures from the experimental measurements. These deviations arise from a combination of physical processes (breaking, associated turbulence and nonlinear wave–wave interactions) and model-related limitations (e.g. grid-dependent dissipation in OpenFOAM and the parametrised breaking dissipation in OW3D), rather than from purely numerical error. OpenFOAM is able to resolve spilling breaking events, with locally high crest-front velocities and enhanced high-wavenumber content in both the free surface and the kinematics, whereas OW3D cannot represent overturning or air entrainment and instead applies local smoothing based on a vertical-acceleration threshold. As a result, the models diverge most clearly at times and locations of breaking, but remain broadly consistent elsewhere.

The dispersion analyses indicate that, for non-breaking conditions, both models reproduce the linear dispersion relation well over the energetic part of the spectrum. OW3D maintains negligible dissipation for the non-breaking cases, whilst OpenFOAM and the experiments typically under-produce the target significant wave height by up to around 10–15%, particularly for the lower-period sea states where there are a larger number of wavelengths. The spatial variation of $H_{m0}$ also highlights practical limitations: in the physical basin, imperfect absorption and the curved reflected wave field mean that incident–reflected separation is most reliable near the reflection gauge array around x=0, and some residual reflected energy persists away from this region. This also highlights broader challenges of separating reflections in nonlinear sea states.

Overall, the results confirm that a high-order FNPF model such as OW3D is highly efficient and accurate for broadbanded and nonlinear sea states. It captures surface-elevation statistics, kinematics, dispersion, and dissipation with good fidelity, other than at times/locations of breaking, at a tiny fraction of the computational cost of VoF CFD. By contrast, the OpenFOAM VoF–LES model provides a more complete description of the fluid and is able to resolve overturning free surfaces and the larger scale turbulent motions associated with breaking. It can be more naturally extended to include complex fixed or floating structures. However, these advantages come at the expense of lower formal numerical order, higher sensitivity to grid and timestep choices, and a computational cost that is several orders of magnitude larger for the present 2D configurations.

To summarise, for applications dominated by non-breaking wave propagation, for estimating statistics of surface elevation and kinematics, and for studies that require many irregular realisations or long-duration simulations, a high-order FNPF model such as OceanWave3D is strongly preferred. For applications where local breaking processes, impact pressures, air entrainment, or detailed wave–structure interaction are critical, a VoF CFD approach such as OpenFOAM, or an equivalent Navier–Stokes solver, is more appropriate despite the higher cost. In many realistic three-dimensional offshore applications, particularly those involving directionally spread breaking waves and complex structures, a coupled framework that combines FNPF and CFD tools is likely to offer the best compromise between accuracy and efficiency, and this will be the focus of future work.

## Figures and Tables

**Fig. 21:**
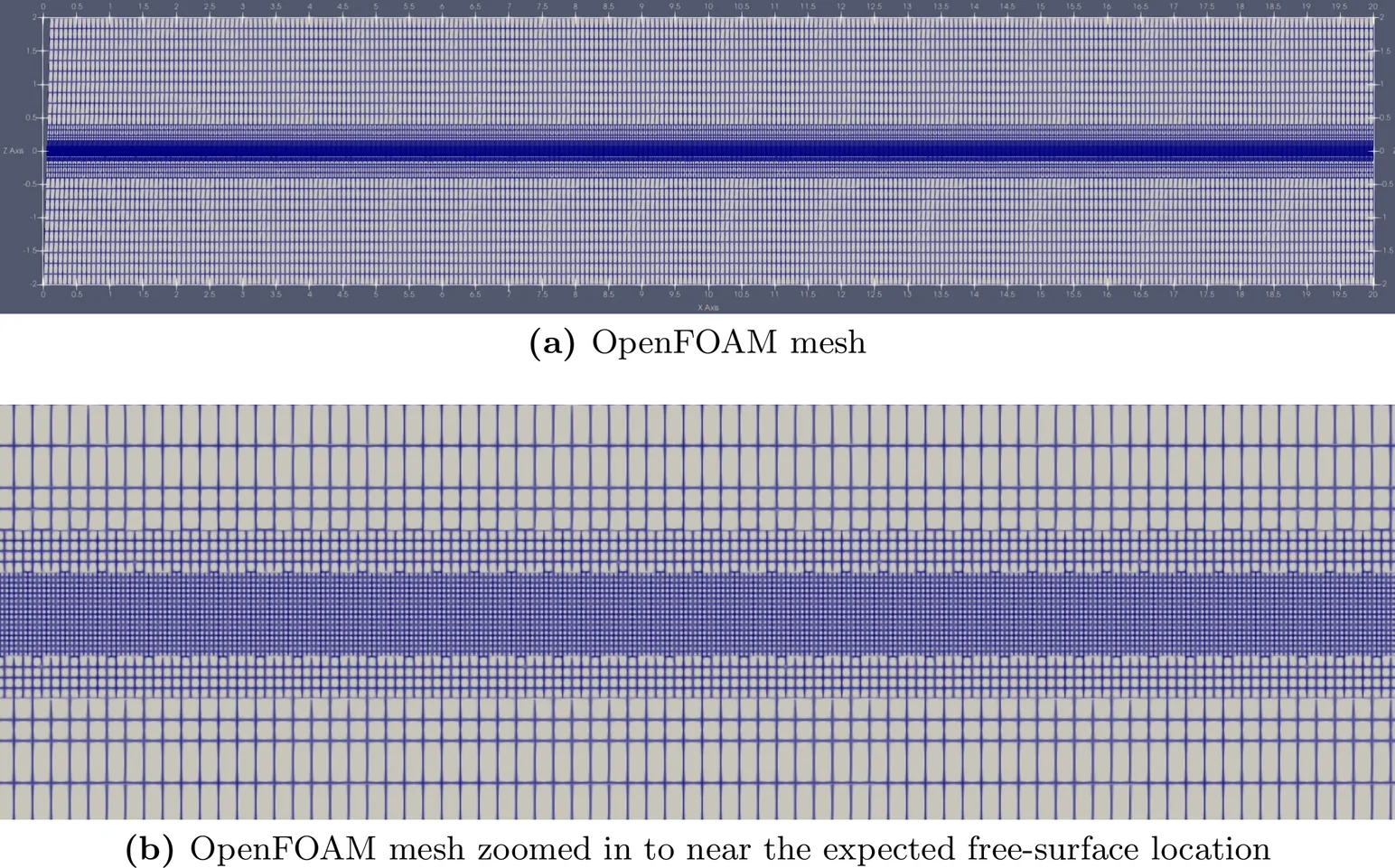
OpenFOAM mesh and zoom near the expected free-surface location

**Fig. 22:**
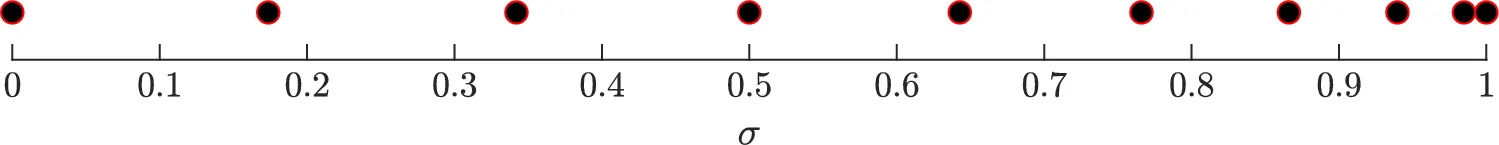
Values of sigma used for OceanWave3D: [0, 0.174, 0.342, 0.5, 0.643, 0.766, 0.866, 0.940, 0.985, 1]

**Fig. 23:**
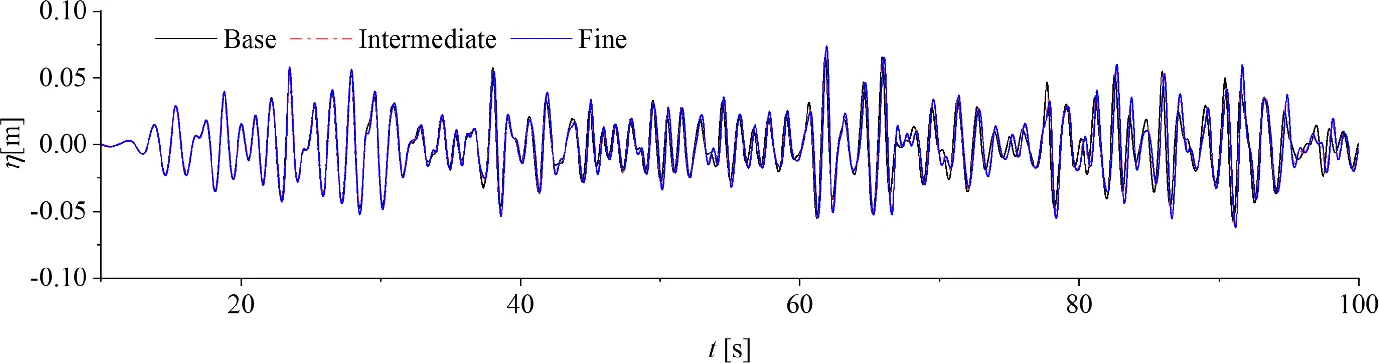
Mesh sensitivity for case 4. Results are shown for a base mesh (Δx=0.03125 m), intermediate mesh (Δx=0.0156 m), and fine mesh (Δx=0.011 m)

**Fig. 24:**
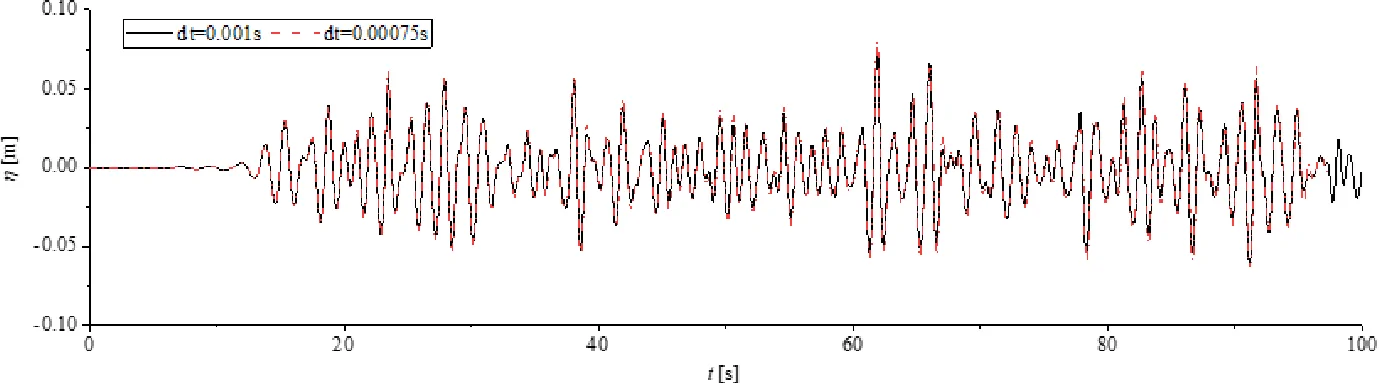
Timestep sensitivity for case 4 (same gauge location and analysis window as in the main results)

**Fig. 25:**
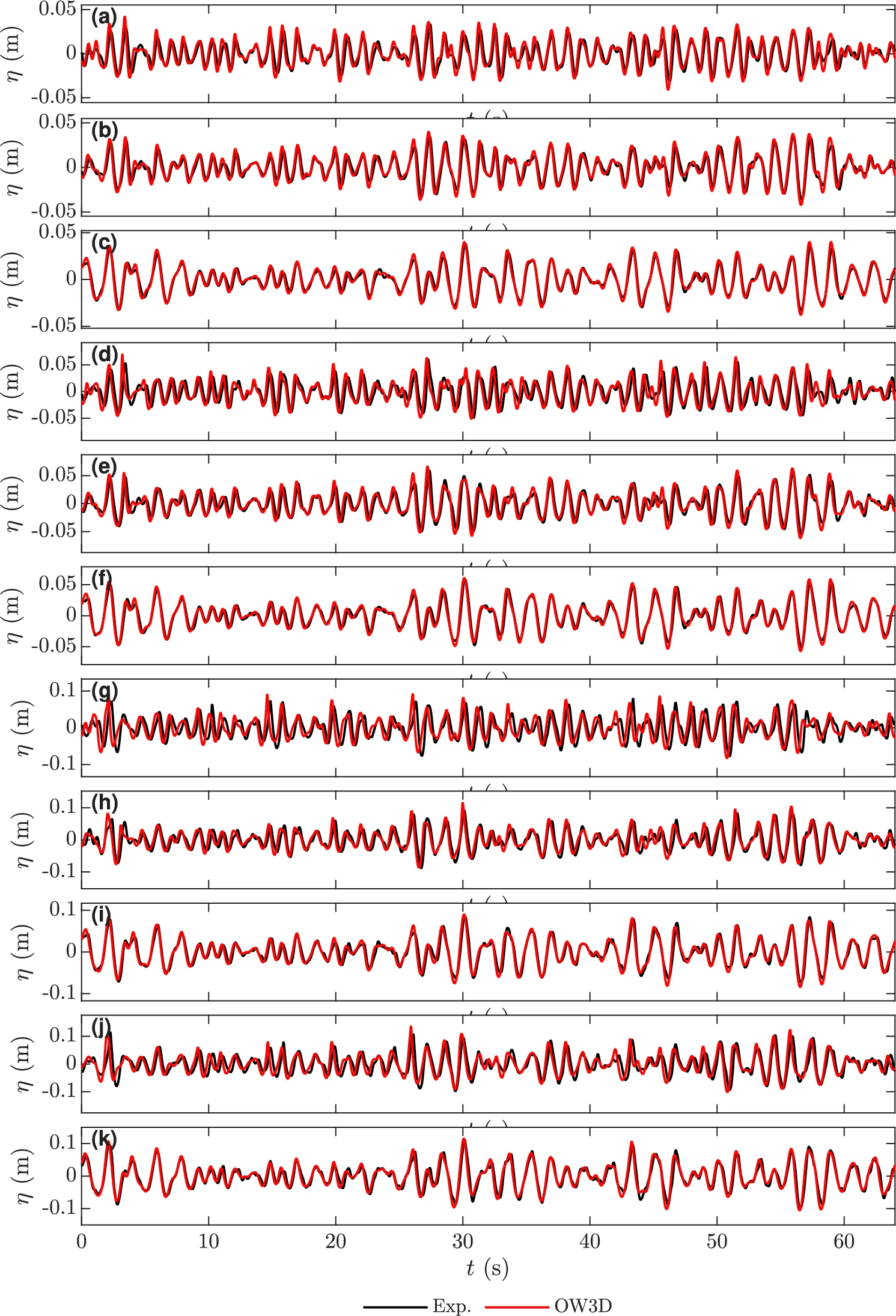
Time-series comparisons for all 11 cases between experimental measurements and OceanWave3D outputs at the target position *x* = 0 m. Data is presented without applying a phase shift. Panels (a)–(k) correspond to cases (1)–(11) in Table 1, respectively

**Fig. 26:**
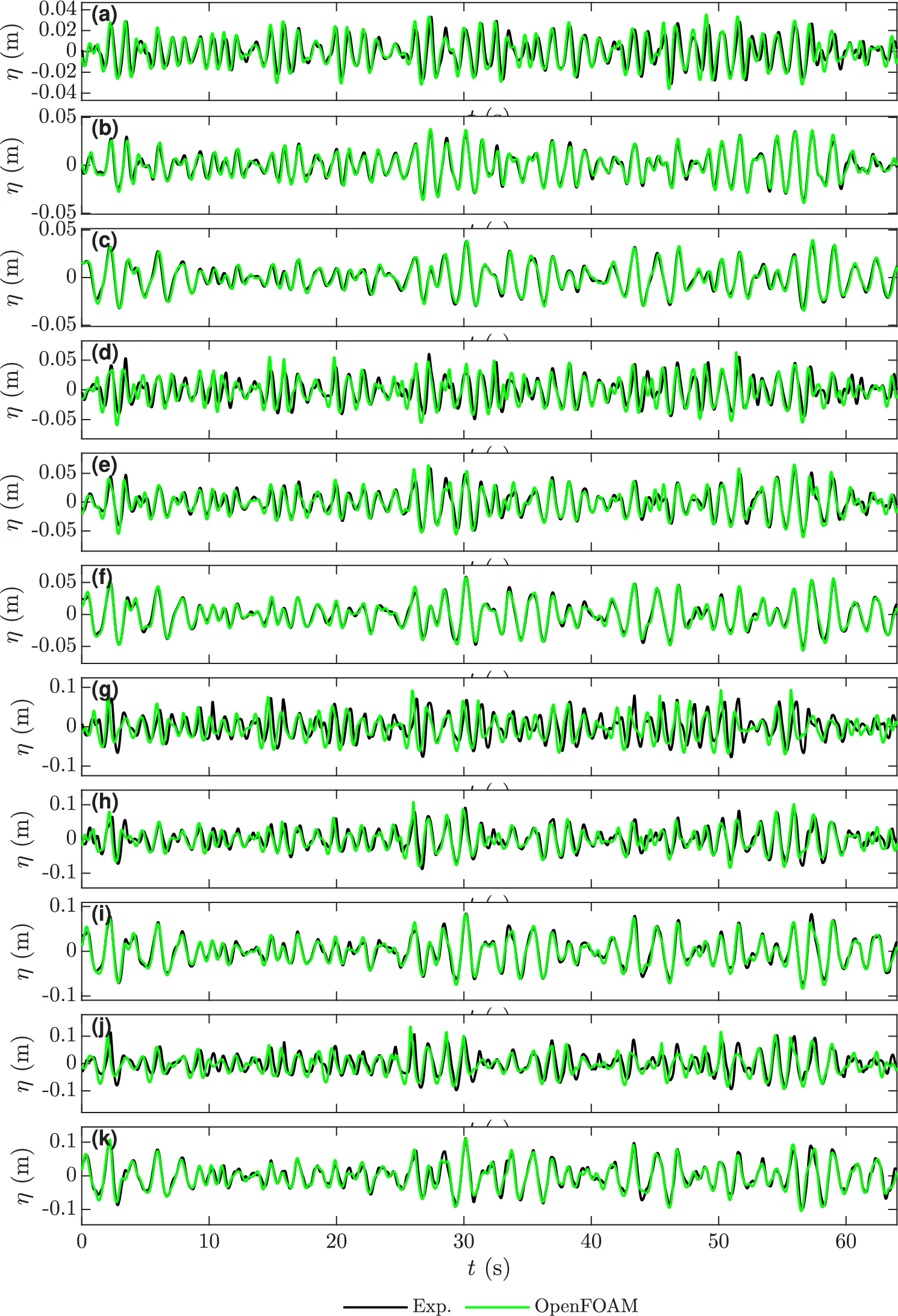
Time-series comparisons for all 11 cases between experimental measurements and OpenFOAM outputs at the target position *x* = 0 m. Data is presented without applying a phase shift. Panels (a)–(k) correspond to cases (1)–(11) in Table 1, respectively

**Fig. 27:**
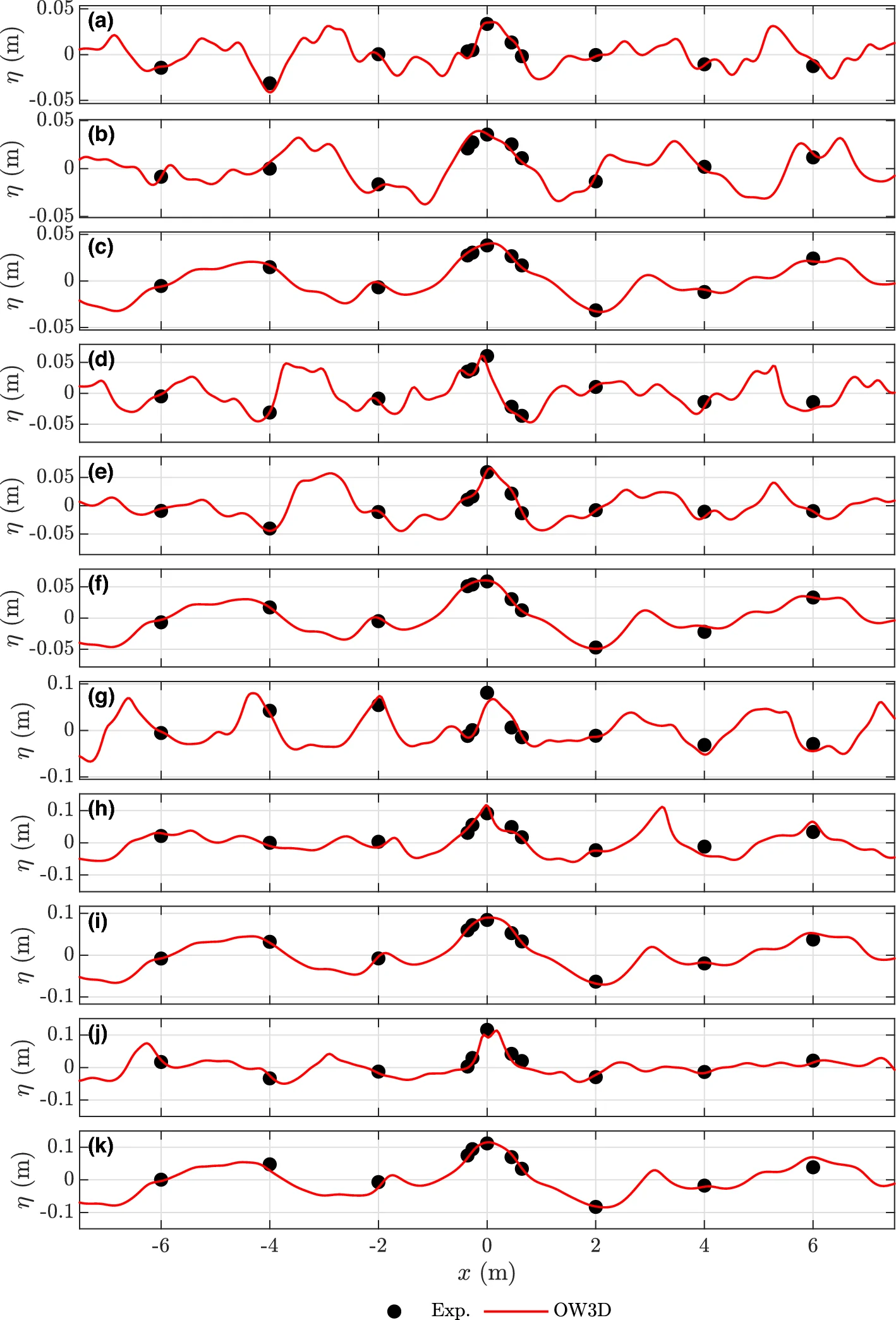
Spatial comparisons at time of maximum in experiments at *x* = 0 m for all 11 cases between experimental measurements and OceanWave3D outputs. Data is presented after phase shift applied. Panels (a)–(k) correspond to cases (1)–(11) in Table 1, respectively

**Fig. 28:**
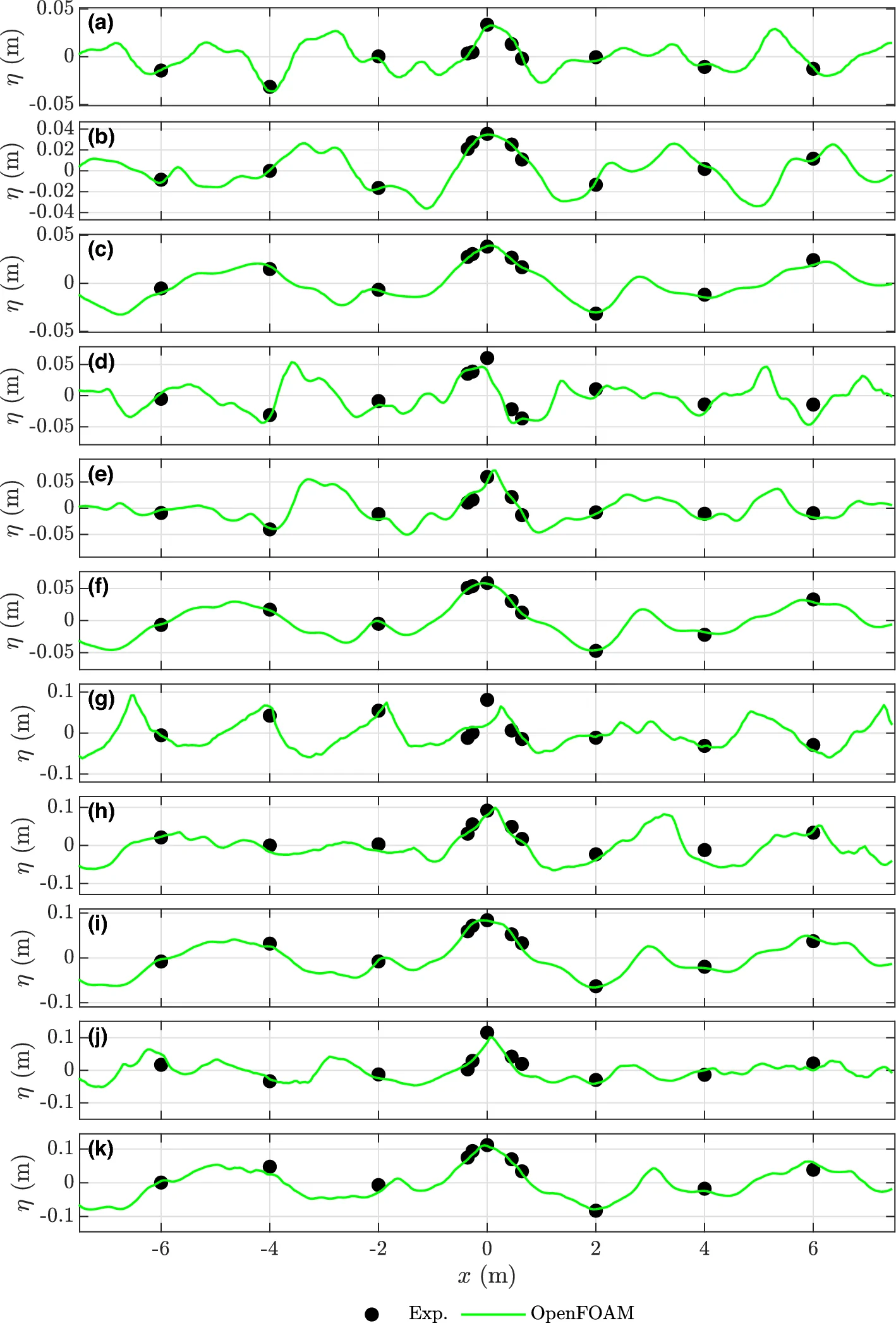
Spatial comparisons at time of maximum in experiments at *x* = 0 m for all 11 cases between experimental measurements and OpenFOAM outputs. Data is presented after phase shift applied. Panels (a)–(k) correspond to cases (1)–(11) in Table 1, respectively

## Data Availability

Experimental and numerical model output data available on request.

## Reflection analysis

This appendix quantifies residual reflections in the experiments and numerical wave tanks using linear frequency-domain decomposition (Zelt and Skjelbreia 1993). For each case, the surface elevation record is decomposed into incident and reflected components and their associated energy spectra, $S_i(f)$ and $S_r(f)$. We then define an integrated reflection coefficient

A1 $$\begin{aligned} K_r = \sqrt{\frac{\sum _{f_{\min }\le f \le f_{\max }} S_r(f)\,\Delta f}{\sum _{f_{\min }\le f \le f_{\max }} S_i(f)\,\Delta f}}, \end{aligned}$$

which is equivalent to the ratio of RMS amplitudes associated with the reflected and incident components over the chosen frequency band. Here $f_{\min }=0.2$ Hz and $f_{\max }=2.0$ Hz reflecting the frequency band available for wave generation in the FloWave basin, and the summations are taken over the discrete FFT frequency bins. The resulting values of $K_r$ for all cases are shown in Fig. 19. For the steepest cases, $K_r$ should be interpreted as an effective indicator under linear assumptions, since nonlinear bound-wave content and breaking will violate the assumptions underlying linear separation.

To confirm that the main differences between models and experiments are not associated with the reflection treatment (reflections removed from experiments but not the models), we present $r^2$ values for surface elevations at x=0 m with reflections removed from all data, once a bulk phase shift has been applied. This is shown in Fig. 20 and is the equivalent plot to Fig. 5 (where reflections were not removed from numerical model outputs).

## Grid information

This appendix provides mesh and vertical coordinate details for reproducibility. Figure. 21 shows the OpenFOAM mesh used in the simulations, and Fig. 22 defines the σ-coordinate distribution used in OceanWave3D.

## Discretisation sensitivity

As described in Sect. 2.4, larger time steps and coarser spatial resolution led to excessive numerical dissipation in OpenFOAM (see Fig. 18). In this appendix, we therefore assess sensitivity of the predicted free-surface elevation to the spatial grid resolution (Fig. 23) and timestep (Fig. 24). The comparisons are made for a representative irregular case (case 4) at the target gauge location used in the main results.

**Table 3:** Bulk phase offsets (lags) used to align model time series with experiments at x=0

Case	$\textrm{lag}_{\textrm{OW3D}}$	$\Delta t_{\textrm{OW3D}}$ (s)	$\textrm{lag}_{\textrm{OF}}$	$\Delta t_{\textrm{OF}}$ (s)
	(samples)		(samples)	
1	-3	−0.09375	-3	−0.09375
2	-3	−0.09375	1	0.03125
3	-2	−0.06250	1	0.03125
4	-5	−0.15625	-4	−0.12500
5	-3	−0.09375	-2	−0.06250
6	-2	−0.06250	0	0.00000
7	-6	−0.18750	-6	−0.18750
8	-4	−0.12500	-3	−0.09375
9	-2	−0.06250	-1	−0.03125
10	-5	−0.15625	-5	−0.15625
11	-2	−0.06250	-1	−0.03125

Figure 23 compares three meshes, where the horizontal spacing is varied whilst maintaining the same numerical setup and physical parameters. The coarsest mesh shows slightly stronger damping of the wave signal, consistent with increased numerical dissipation. In contrast, the intermediate (Δx=0.0156 m mesh used throughout the manuscript) and fine meshes yield very similar surface-elevation time series at the target location, indicating that the primary surface-elevation predictions are insensitive to further mesh refinement for this case.

Figure 24 presents an analogous sensitivity test for the timestep. Larger timesteps again lead to increased dissipation and reduced crest elevations (Fig. 18), whilst the selected timestep (0.001 s) used in the main study provides stable predictions and minimal change relative to the smaller timestep result for this representative case. These tests support the discretisation choices adopted in Sect. 2.4 for the present comparison study.

## Time-series and spatial comparisons for all sea states

This appendix provides the full set of time series and spatial comparisons for all 11 sea states. Figures 25 and 26 present time-series comparisons at x=0, and Figs. 27 and 28 present the corresponding spatial profiles at the time of maximum recorded during experiments.

## Bulk phase offsets

For each case, we estimate a bulk phase offset by maximising the cross-correlation between the model and experimental surface-elevation time series at x=0 (when resampled onto a consistent base frequency of $f_s=32$ Hz). Table 3 reports the corresponding lag in both integer samples along with the equivalent time shift $\Delta t=\textrm{lag}/f_s$. A positive lag indicates that the model time series lags the experiment (model is delayed), whilst a negative lag indicates that the model leads the experiment
